# Soft tissue tumor imaging in adults: European Society of Musculoskeletal Radiology-Guidelines 2023—overview, and primary local imaging: how and where?

**DOI:** 10.1007/s00330-023-10425-5

**Published:** 2023-12-07

**Authors:** Iris-Melanie Noebauer-Huhmann, Filip M. Vanhoenacker, Joan C. Vilanova, Alberto S. Tagliafico, Marc-André Weber, Radhesh K. Lalam, Thomas Grieser, Violeta Vasilevska Nikodinovska, Jacky W. J. de Rooy, Olympia Papakonstantinou, Catherine Mccarthy, Luca Maria Sconfienza, Koenraad Verstraete, José Martel-Villagrán, Pavol Szomolanyi, Frédéric E. Lecouvet, Diana Afonso, Omar M. Albtoush, Giacomo Aringhieri, Remide Arkun, Gunnar Aström, Alberto Bazzocchi, Rajesh Botchu, Martin Breitenseher, Snehansh Chaudhary, Danoob Dalili, Mark Davies, Milko C. de Jonge, Berna D. Mete, Jan Fritz, Jan L. M. A. Gielen, Geoff Hide, Amanda Isaac, Slavcho Ivanoski, Ramy M. Mansour, Lorenzo Muntaner-Gimbernat, Ana Navas, Paul O´Donnell, Şebnem Örgüç, Winston Rennie, Santiago Resano, Philip Robinson, Hatice T. Sanal, Simone A. J. Ter Horst, Kirsten van Langevelde, Klaus Wörtler, Marita Koelz, Joannis Panotopoulos, Reinhard Windhager, Johannes L. Bloem

**Affiliations:** 1 Department of Biomedical Imaging and Image Guided Therapy, Division of Neuroradiology and Musculoskeletal Radiology, Medical University of Vienna, Vienna, Austria; 2 Department of Radiology AZ Sint Maarten Mechelen, University Hospital Antwerp, Antwerp, Belgium; 3 Faculty of Medicine and Health Sciences, University of Ghent, Ghent, Belgium; 4 Department of Radiology, Clínica Girona, Institute of Diagnostic Imaging (IDI) Girona, University of Girona, Girona, Spain; 5 Department of Health Sciences (DISSAL), University of Genoa, Genoa, Italy; 6 Department of Radiology, IRCCS Ospedale Policlinico San Martino, Genoa, Italy; 7 Institute of Diagnostic and Interventional Radiology, Pediatric Radiology and Neuroradiology, University Medical Center Rostock, Rostock, Germany; 8 Department of Radiology, Robert Jones and Agnes Hunt Orthopaedic Hospital, Oswestry, UK; 9 Dept. for Diagnostic and Interventional, Radiology University Hospital Augsburg, Augsburg, Germany; 10 Medical Faculty, Ss. Cyril and Methodius University, Skopje, Macedonia; 11 Department of Radiology, University Surgical Clinic “St. Naum Ohridski” Skopje, Skopje, Macedonia; 12 Department of Imaging, Radiology, Radboud University Medical Center, Nijmegen, The Netherlands; 13 2Nd Department of Radiology, Attikon Hospital, National and Kapodistrian University of Athens, Athens, Greece; 14 Oxford Musculoskeletal Radiology and Oxford University Hospitals, Oxford, UK; 15 IRCCS Istituto Ortopedico Galeazzi, Milan, Italy; 16 Dipartimento Di Scienze Biomediche Per La Salute, Università Degli Studi Di Milano, Milan, Italy; 17 Department of Radiology, Ghent University Hospital, Ghent, Belgium; 18 Radiology Department, Hospital Universitario Fundación Alcorcón, Madrid, Spain; 19 High Field MR Center, Department of Biomedical Imaging and Image-Guided Therapy, Medical University Vienna, Vienna, Austria; 20 Department of Imaging Methods, Institute of Measurement Science, Slovak Academy of Sciences, Bratislava, Slovakia; 21 Department of Radiology and Medical Imaging, Cliniques Universitaires Saint Luc, Institut de Recherche Expérimentale et Clinique (IREC), Université Catholique de Louvain (UCLouvain), Brussels, Belgium; 22 Hospital Particular da Madeira, and Hospital da Luz Lisboa, Lisbon, Portugal; 23 Department of Radiology, University of Jordan, Ammam, Jordan; 24 Academic Radiology, Department of Translational Research and New Technologies in Medicine and Surgery, University of Pisa, Pisa, Italy; 25 Ege University Medical School Izmir, Izmir, Turkey Star Imaging Center Izmir, Izmir, Turkey; 26 Department of Immunology, Genetics and Pathology (Oncology) and Department of Surgical Sciences (Radiology), Uppsala University, Uppsala, Sweden; 27 Diagnostic and Interventional Radiology, IRCCS Istituto Ortopedico Rizzoli, Bologna, Italy; 28 Department of Musculoskeletal Radiology, Royal Orthopedic Hospital, Birmingham, UK; 29 Sigmund Freud Privatuniversität, Vienna, Austria; 30 Oxford University Hospitals NHS Foundation Trust, Oxford, UK; 31 Academic Surgical Unit, South West London Elective Orthopaedic Centre (SWLEOC), London, UK; 32 Department of Radiology, St. Antonius Hospital, Utrecht, The Netherlands; 33 Department of Radiology School of Medicine, Izmir Demokrasi University, Izmir, Turkey; 34 Department of Radiology, NYU Grossman School of Medicine, New York, USA; 35 Diagnostic and Interventional Radiology, Eberhard Karls University Tuebingen, University Hospital Tuebingen, Tübingen, Germany; 36 Department of Radiology and Medical Imaging, University Hospital Antwerp, Edegem, Belgium; 37 Department of Radiology, Freeman Hospital, Newcastle Upon Tyne, UK; 38 School of Biomedical Engineering and Imaging Sciences, King’s College London, London, UK; 39 St. Erasmo Hospital for Orthopaedic Surgery and Traumatology Ohrid, Ohrid, Macedonia; 40 Oxford University Hospitals, Oxford, UK; 41 Hospital Univeritario Son Espases Balearic Islands University, Palma, Spain; 42 Department of Radiology, Division of Musculoskeletal Radiology, Leiden University Medical Center, Leiden, The Netherlands; 43 Royal National Orthopaedic Hospital, Stanmore, UK; 44 Manisa Celal Bayar University, Manisa, Turkey; 45 Clinical MSK Radiology, Loughborough University, Leicester Royal Infirmary, Leicester, UK; 46 Hospital Universitario Ramón y Cajal, Madrid, Spain; 47 Musculoskeletal Radiology Department Chapel Allerton Hospital, Leeds Teaching Hospitals NHS Trust, Leeds, UK; 48 NIHR Leeds Biomedical Research Centre, Leeds, UK; 49 Radiology Department, University of Health Sciences, Gülhane Training and Research Hospital, Istanbul, Turkey; 50 Princess Máxima Center for Pediatric Oncology, Utrecht, The Netherlands Department of Radiology and Nuclear Medicine, University Medical Centre Utrecht, Utrecht, The Netherlands; 51 Department of Radiology, Leiden University Medical Center, Leiden, The Netherlands; 52 Musculoskeletal Radiology Section, Klinikum Rechts der Isar, Technical University of Munich - TUM School of Medicine, Munich, Germany; 53 Clinical Institute of Pathology, Medical University of Vienna, Vienna, Austria; 54 Departement of Orthopaedics and Traumatology, Division of Orthopaedics, Medical University of Vienna, Vienna, Austria; 55 Departement of Orthopaedics and Traumatology, Medical University of Vienna, Vienna, Austria; 56 Department of Radiology, LUMC, Leiden, The Netherlands

**Keywords:** Practice guideline, Consensus, Neoplasms, Connective and soft tissue, Diagnostic imaging

## Abstract

**Objectives:**

Early, accurate diagnosis is crucial for the prognosis of patients with soft tissue sarcomas. To this end, standardization of imaging algorithms, technical requirements, and reporting is therefore a prerequisite. Since the first European Society of Musculoskeletal Radiology (ESSR) consensus in 2015, technical achievements, further insights into specific entities, and the revised WHO-classification (2020) and AJCC staging system (2017) made an update necessary. The guidelines are intended to support radiologists in their decision-making and contribute to interdisciplinary tumor board discussions.

**Materials and methods:**

A validated Delphi method based on peer-reviewed literature was used to derive consensus among a panel of 46 specialized musculoskeletal radiologists from 12 European countries. Statements were scored online by level of agreement (0 to 10) during two iterative rounds. Either “group consensus,” “group agreement,” or “lack of agreement” was achieved.

**Results:**

Eight sections were defined that finally contained 145 statements with comments. Overall, group consensus was reached in 95.9%, and group agreement in 4.1%. This communication contains the first part consisting of the imaging algorithm for suspected soft tissue tumors, methods for local imaging, and the role of tumor centers.

**Conclusion:**

Ultrasound represents the initial triage imaging modality for accessible and small tumors. MRI is the modality of choice for the characterization and local staging of most soft tissue tumors. CT is indicated in special situations. In suspicious or likely malignant tumors, a specialist tumor center should be contacted for referral or teleradiologic second opinion. This should be done before performing a biopsy, without exception.

**Clinical relevance:**

The updated ESSR soft tissue tumor imaging guidelines aim to provide best practice expert consensus for standardized imaging, to support radiologists in their decision-making, and to improve examination comparability both in individual patients and in future studies on individualized strategies.

**Key Points:**

*• Ultrasound remains the best initial triage imaging modality for accessible and small suspected soft tissue tumors.*

*• MRI is the modality of choice for the characterization and local staging of soft tissue tumors in most cases; CT is indicated in special situations. Suspicious or likely malignant tumors should undergo biopsy.*

*• In patients with large, indeterminate or suspicious tumors, a tumor reference center should be contacted for referral or teleradiologic second opinion; this must be done before a biopsy.*

**Supplementary information:**

The online version contains supplementary material available at 10.1007/s00330-023-10425-5.

## Introduction

Soft tissue sarcomas are rare, and comprise a heterogeneous group of entities [1], leading to diagnostic challenges. An early, accurate diagnosis is crucial for the prognosis of these patients. At the same time, clinical infrastructure differs considerably throughout Europe. The same is true for the attitudes towards the use of advanced imaging techniques. This results in notable variability in clinical practice.

Since the first consensus on soft tissue tumor imaging in adults of the European Society of Musculoskeletal Radiology (ESSR) in 2015, technical achievements, further insights into specific entities, the revised WHO-classification (2020) [1], and a new version of the American Joint Committee on Cancer (AJCC) staging system (2017) [2] made an update of the ESSR consensus guidelines necessary [3]. A Delphi process [4], evidence based on current literature where possible, enables to derive consensus on complex problems among a panel of experts [5], and has been used by the ESSR elsewhere recently [6].

The updated ESSR agreement for imaging of soft tissue tumor aims to provide best practice expert consensus guidelines for standardized imaging algorithms, techniques, and reporting in soft tissue tumors of adults. These recommendations are intended to support radiologists in their decision-making when first being confronted with a suspected soft tissue tumor and help them in their contribution to interdisciplinary tumor board meetings. Standardization can also be useful for follow-up in the individual patient, as comparison of serial examinations even when performed in different institutions can be compared reliably. Finally, standardized examinations may provide better databases for multicenter studies. Standardization may also facilitate evaluations of large datasets for optimization of individualized care.

## Materials and methods

A validated Delphi method [5–9] on the base of peer-reviewed literature was used to derive consensus among a panel of 46 specialized musculoskeletal radiologists from 12 European countries, all being members of the tumor subcommittee of the ESSR. Institutional review board approval was not required for the present consensus as patients were not involved. Major sections were defined. For each section, working groups provided statements with comments, based on the current literature, following a search on PubMed and the Cochrane Library. The statements were validated by two orthopedic tumor surgeons and one pathologist specialized in musculoskeletal tumors. All statements were imported into an online questionnaire, using the online platform Google Forms® [10]. The panel members were then asked to score their level of agreement with each statement, on a scale from 0 to 10, with 10 being the highest grade of agreement. Minimum statement scoring by the panel was considered if a median of at least 8 and an interquartile range of less than 4 were achieved. For the statements which fulfilled these criteria, the level of agreement was calculated, and assigned as either “group consensus,” “group agreement,” or “lack of agreement.” “Group consensus” was defined as at least 80% of respondents scoring at least 8, “Group agreement” was defined as 67–79% of respondents scoring at least 8. “Lack of agreement” was assigned if the previous conditions were not met. Respondents also had the opportunity to make suggestions for altering the statements for future rounds of voting, especially if they disagreed with the statements.

A face-to-face meeting of the panelists was organized on the occasion of the ESSR congress in 2022, where open questions on the Delphi process were addressed, the preliminary results of round 1 were presented, and specific comments were discussed. After the meeting, round 1 was re-opened for further ratings. After termination of round 1, the statements without group consensus, and all statements with suggestions for any change were modified appropriately by the organizing panelist (I.-M. N.-H.). Additional statements were added as suggested. All these revised statements were circulated and further amended. All statements which had been changed since round 1 were then provided online for scoring in a second iterative questionnaire round. The results of round two were re-calculated and labeled as described for round 1. After round 2, the rating was terminated for each statement.

## Results

Eight sections were defined that finally contained 145 statements overall. After round 2, all statements had reached either group consensus or group agreement. Group consensus was reached in 139/145 statements (95.9%), and group agreement was achieved in 6/145 statements (4.1%). None of the statements resulted in lack of agreement.

The first two of the eight sections included (1) primary diagnosis of soft tissue tumors, with background information and local imaging (62 statements, with 61 of them with group consensus, 1 with group agreement, and none with lack of agreement); (2) the role of referring hospitals and tumor reference centers (12 statements, 12/0/0, respectively). The statements of these two sections are described in detail in this part of the consensus (part I).

The remaining six sections will be published subsequently and will deal with whole-body staging in sarcoma at the time of primary diagnosis, non-malignant entities that require special management, pitfalls, and special aspects in soft tissue tumor imaging, imaging during and immediately after neoadjuvant therapy in soft tissue sarcoma, and post-therapeutic follow-up in sarcoma.

**Fig. 1:**
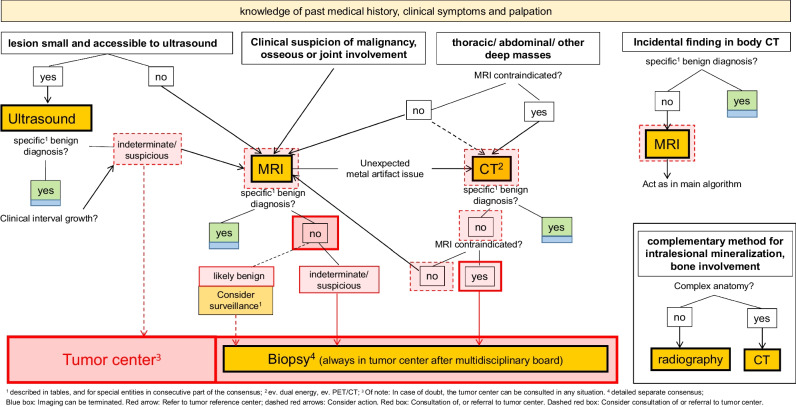
Primary diagnosis: algorithm for local imaging of soft tissue mass

The first section of part I, primary diagnosis of soft tissue tumors, covers local imaging algorithms; Fig. 1 gives an overview. Statements and their level of agreement are provided in Table 1. They deal with background information requirements such as the past medical history (PMH) and the clinical situation. Updated detailed recommendations for an optimized soft tissue tumor imaging algorithm and technical requirements are also provided. Standards for reporting, now with a detailed checklist for clinical routine use, have been developed and can be found as supplementary material (figure S1). Recommendations relating to the role of guidelines and tumor reference centers, corresponding to the second section, are provided in Table 2, and are also addressed by Fig. 1. For all statements, comments with references are provided in the electronic supplementary material.

**Table 1:** Section 1. Primary diagnosis, local imaging. Statements

	Median, IQR (difference interval), Level of agreement
**1.1 History and Physical Examination (“H & P”):**	
**1.1.1. Regarding the past medical history (PMH) of the patient**, the following information should be available for the radiologist:	10; 0,5 (9.5–10); 97%
-When did the patient first notice the lesion?	
-Does it change in size? Is it growing, and how fast?	
-Has there been a recent trauma? Is the patient anticoagulated?	
-Is there any oncologic history of the patient?	
-Is there a family history of tumors or syndromes?	
-Is there history of previous surgery or of radiation therapy?	
A standardized checklist, primarily filled out by the patient, and discussed with the radiologist, is considered advisable. The patient or the referring clinician should also be asked to provide previous imaging if available	10; 1 (9–10); 91%
**1.1.2. Regarding clinical symptoms and palpation**, the following information should be available for the radiologist:	10; 1 (9–10); 97%
-Is the lesion palpable, and if so, is it hard or soft?	
-Is it movable against the skin and underlying tissue?	
-Is the lesion painful? Tinel sign?	
-Are there skin alterations or pathologic vessels?	
-Single or multiple lesions?	
**1.2 Imaging modalities and algorithm:**	
**1.2.1. Role of Ultrasound:**	
-Ultrasound (US) is considered the appropriate initial triage imaging modality for a suspected soft tissue tumor, if accessible by US and small (< 5 cm). When US diagnosis is not typical for a diagnosis, refer to Magnetic Resonance Imaging (MRI) or even biopsy*	10; 1.5 (8.5–10); 88%
*Caveat: MRI should be performed prior to biopsy (if it will add to lesion characterization), not afterwards	10; 1 (9–10); 96%
-Benign lesions that can be diagnosed on US include:	10; 1 (9–10); 91%
-simple cyst, bursa, synovial/ganglion cyst (purely cystic well-defined lesion without any solid component, anechoic, with posterior acoustic enhancement and no internal vascularity)	
-superficial lipoma (homogeneous well defined, often encapsulated, and compressible with no clinical concern, clinically stable, < 10 cm and with documented stability on US (at least 6 months follow-up)),	
-foreign body granuloma with a compatible history,	
-superficial fibromatosis (e.g., palmar and plantar fibromatosis, infantile digit fibromatosis),	
-muscle hernia and	
-Morton neuroma	
-Benign lesions that can often be diagnosed on US include aneurysms and muscle tears. In any case of doubt, MRI should be performed	10; 1 (9–10); 100%
-Small, superficial soft tissue masses that are likely to be benign, or which have been diagnosed with US (see above) but show interval growth should undergo biopsy (in lesions < 2–3 cm, excisional biopsy can be considered)	10; 1 (9–10); 91%
**1.2.2. Role of Magnetic Resonance Imaging:**	
-MRI is the imaging technique of choice for characterization and local staging of musculoskeletal soft tissue masses with indeterminate ultrasound features and large tumors	10; 0 (10–10); 97%
-Primary MRI should be considered instead of US if there is a clinical suspicion of malignancy, if the mass is deep, rapidly enlarging, and if there is osseous or joint involvement	10; 0 (10–10); 97%
-The following lesions can/may be reliably characterized by MRI:	9.5; 1 (9–10); 91%
-Anatomic variations, vascular malformation (+ high flow/low flow)	
-ganglion cyst, Baker cyst, bursitis	
-Lipoma, peripheral nerve sheath tumor (neurofibroma/schwannoma, apart from “ancient”schwannoma), TSGCT/pigmented villonodular synovitis (PVNS)	
-hematoma, muscle tear, myositis ossificans, and aneurysm	
**1.2.3. Role of Projection Radiography:**	
-There is limited role of radiographs in local staging of soft tissue sarcoma. However, radiography is a complementary modality for the identification and characterization of (a) intralesional mineralization patterns and (b) potential bone involvement of soft-tissue masses	10; 1 (9–10); 97%
**1.2.4. Role of Computed Tomography:**	
-For regions with a complex anatomy (e.g., axial skeleton, head/ neck, thoracic, and pelvic areas), CT is preferred over radiography	10; 1 (9–10); 94%
-In cases where metallic structures cause unacceptable artifacts on MRI, even though modern metal artifact reduction techniques are applied, the use of CT with metal artifact reduction protocols may be useful	10; 1 (9–10); 95%
-A deep soft tissue mass discovered incidentally during body CT requires further diagnosis. Depending on the lesion morphology, either MRI or immediate biopsy may be indicated	9; 2 (8–10); 88%
-CT can be considered instead of MRI for complex thoracic/ abdominal / other deep masses. CT should be performed in case of complex thoracic/ abdominal / other deep masses if MRI is unavailable or contraindicated	9.5; 1.5 (8.5–10); 94%
-Dual energy CT scan (DECT) can aid in metallic artefact reduction as well as in evaluation of soft tissue calcification	9; 1.5 (8.5–10); 84%
**1.2.5. Role of other techniques:**	
-There is no role of bone scintigraphy in local staging of soft tissue sarcoma	10; 1 (9–10); 91%
-There is no role of PET-MRI and MRS in routine local staging of soft tissue sarcoma	10; 1 (9–10); 91%
**1.3 Imaging technique:**	
**1.3.1. Ultrasound technique:**	
-The recommended frequency is at least 12 MHz. Lower frequencies can be used for detection of deeper lesions	9; 2 (8–10); 94%
-Contrast enhanced Ultrasound (CEUS) can be considered by radiologists with special experience in CEUS for biopsy guidance in large lesions	10; 2 (8–10); 87%
-Elastography is not considered necessary	10; 1 (9–10); 88%
-Evidence regarding the application of ultrasound elastography for differentiating benign from malignant MSK soft tissue tumors is conflicting. There is no significant proof to recommend ultrasound elastography as a method for identification of MSK soft-tissue tumor malignancy. Shearwave elastography is a feasible technique for evaluation of benign MSK soft tissue tumors, with insufficient proof to be recommend as an imaging method for their differentiation	10; 1 (9–10); 94%
**1.3.2. MRI technique:**	
-The recommended field strength of the MRI scanner for soft tissue tumors is at least 1.5 T. 3 T may be useful and is optimal for advanced imaging such as spectroscopy	10; 1 (9–10); 100%
-A cutaneous marker should be applied	10; 1 (9–10); 97%
-The field of view should be as large as necessary to image the entire lesion, peritumoral oedema, and a layer of adjacent normal tissue, and to image nonpalpable masses reliably	10; 1 (9–10); 97%
-The voxel size should be as low as feasible to demonstrate relevant morphologic features and anatomic detail	10; 1 (9–10); 100%
-The size of the tumor should be measured in three dimensions	10; 0 (10–10); 100%
-Axial sequences with high spatial resolution are important to define tumor margins, tissue and compartment involvement, and neurovascular, bone, and joint involvement	10; 1 (9–10); 97%
-The recommended basic protocol includes combination of T1-weighted and a fluid-sensitive, fat-saturated (FS) sequence, both parallel to the long axis of the tumor	10; 1 (9–10); 88%
-The use of Dixon technique for T2w and T1w sequences is advantageous, as a single Dixon based acquisition provides four contrasts, including images with and without fat suppression, and information about the fat content of a lesion (detection on Fat images and quantification on Fat Fraction maps)	9.5; 1 (9–10); 100%
-An axial T2-weighted sequence without fat saturation can provide further information about the tumor matrix	10; 2 (8–10); 85%
-A diffusion weighted sequence (DWI) with calculation of the apparent diffusion coefficient (ADC) may also be useful	8; 3 (7–10); 67%
-The diffusion-weighted sequence of the protocol should have at least two but optimally three b-values ranging from 50 s/mm^2^ to max. 800 or 1000 s/mm^2^	10; 1 (9–10); 95%
-In MRI, intravenous gadolinium contrast administration with the use of dynamic contrast enhanced sequences (DCE) can help in the differentiation of benign versus malignant soft tissue tumors	10; 1 (9–10); 100%
-DCE enables to detect viable intra-tumoral areas and to determine their vascularization patterns, and therefore assists in targeting tumor biopsy	9; 1 (9–10); 87%
-Post Gd subtraction techniques are useful for ruling out areas which present an intrinsically high signal intensity on T1w images (such as melanin or methemoglobin)	10; 1 (9–10); 95%
-When applying appropriate metal artifact reduction MRI techniques, subtraction images of post- and pre- Gd T1w MR images are useful for assessing contrast enhancement next to metallic hardware, which may otherwise be obscured by failed spectral fat suppression	10; 1 (9–10); 100%
**1.3.3. Projection radiography technique**	
- Initial radiographic evaluation should be performed with at least two orthogonal views	10; 0 (10–10); 100%
**1.3.4. CT technique**	
-For the identification and characterization of intralesional mineralization patterns and potential bone involvement, CT without contrast agent application is sufficient	10; 1 (9–10); 94%
-Iodinated contrast agents should be used in cases where CT serves for local staging instead of MRI	10; 1 (9–10); 94%
-In case of metallic hardware, metal artefact reduction algorithms should be used	10; 1 (9–10); 94%
-CT angiography (CTA) can be used for evaluation of the vascular encasement as well as in assessment of suspected tumoral thrombus of encased vessels	10; 2 (8–10); 88%
**1.4 Imaging reports should contain the following information:**	
**1.4.1. Ultrasound:**	
–Anatomical location: Relation to the fascia (superficial, deep), exact anatomical location including compartmental involvement, intra- or intermuscular location, and the relationship to/infiltration of vessels/nerves, and, if possible, joints and/or bone and crucial adjacent structures	10; 1 (9–10); 100%
–Size (in three dimensions; for the method, please see the section below on MRI)	10; 1 (9–10); 100%
–Morphology: borders/margins and shape (with estimation of growth pattern: infiltrative or expansive) and (if possible) presence of a capsule/pseudocapsule; cystic, solid (intralesional echo texture, vascularization (by color-Doppler based Giovagnorio classification), presence or absence of necrosis, bleeding, suspected tumor matrix mineralization)	10; 1 (9–10); 97%
–Concerns about tumor accessibility by US for a definitive diagnosis or the evaluation of local extension	10; 1 (9–10); 100%
–The fact that a lesion is indeterminate in US, with recommendation for subsequent imaging	10; 1 (9–10); 100%
–Change to previous examination/ tumor at the site of a previous excision	10; 1 (9–10); 91%
**1.4.2. MRI (please see also checklist):**	
–Location and 3 D size, MR morphology, shape, border, relation to fascia,	10; 1 (9–10); 100%
–Intra- extracompartmental, relation to adjacent structures (vessels, nerves, joints,…) and surrounding tissue alterations	10; 1 (9–10); 100%
–Distance to external landmark, satellites, multiplicity, locoregional lymph nodes, and other tissue alterations	10; 1 (9–10); 100%
–The image quality should be addressed	10; 0 (10–10); 100%
–Changes to previous images (if available) should be described	10; 0 (10–10); 100%
**1.4.3. Projection radiographs:**	
–Characteristic calcification patterns, bone destruction, and soft tissue swelling	10; 1 (9–10); 100%
–If possible: Density, location, longest diameter	10; 1 (9–10); 100%
–Also important features of unsuspected differential diagnosis	10; 1 (9–10); 100%
–Concerns about superposition effects with, if indicated, recommendation for cross-sectional imaging by CT	10; 1 (9–10); 100%
**1.4.4. CT:**	
–Size/Extension: location, longest diameter, bone (cortical and bone marrow) involvement (destruction/invasion, pressure arrosion/remodelling, sclerosis)	10; 1 (9–10); 97%
–Retroperitoneal liposarcoma: asymmetry in volume and extension of retroperitoneal fat	10; 1 (9–10); 97%
–Morphology: Density/attenuation, patterns of mineralization (e.g., phleboliths, ossification, osteoblastic, chondroid, dystrophic,) and its organization (scattered, peripherally or centrally mature), degree and pattern of vascularity/ contrast enhancement, necrosis	10; 1 (9–10); 97%
–Margin, diffuse surrounding alterations such as stranding and inflammation, free fluid, free air, subsequent alterations of thoracic/abdominal organs (obstruction of ducts, small bowel, …)	10; 1 (9–10); 97%
**1.4.5. PET/CT:** Please see under “whole body staging”

**Table 2:** Section 2. The role of tumor centers. Statements

	Median, IQR (difference interval), Level of agreement
**2.1. Criteria for referral to a sarcoma treatment center:**	
-Any patient with a tumor ≥ 5-cm, or with indeterminate or suspicious US findings, or with clinical suspicion of malignancy	10; 1 (9–10); 95%
-Any patient with indeterminate MRI findings or those suspicious for malignancy	10; 0 (10–10); 97%
-Teleradiologic second opinion workup by a tumor center is appropriate in patients with indeterminate or suspicious MRI findings. It should be offered to the local hospitals in all patients in whom soft tissue sarcoma is suspected	10; 1 (9–10); 90%
**2.2. Examinations that should be performed in a tumor reference center:**	
-The accuracy in tumor characterization may be higher if the MRI is performed and evaluated in a dedicated tumor centre. Where this is not feasible, the MRI scan should be performed as per the technical recommendations of the local tumor center	10; 1 (9–10); 100%
-Patients with suspicion of sarcoma should be referred to the tumor reference center before biopsy or surgery (minimal requirement)	10; 0 (10–10); 100%
**2.3. Role of guidelines:**	
-The guidelines are intended to provide international standards; by publication, and through further promotion by national specialized radiologists, the guidelines will ensure standardization of high-quality soft tissue tumor diagnostic imaging	10; 0 (10–10); 100%
-Radiologists should follow the local tumor center guidelines	10; 0 (10–10); 93%
**2.4. Interdisciplinary tumor team:**	
-Soft tissue tumor board: A multidisciplinary soft tissue sarcoma team should at least include an (orthopedic) tumor surgeon, a musculoskeletal radiologist, a musculoskeletal pathologist, a medical oncologist, and a radiotherapist. Where necessary, other specialists should be invited	10; 1 (9–10); 95%
-An instant discussion between orthopedic tumor surgeon and a musculoskeletal radiologist improves service efficiency and reduces the time to definitive diagnosis	10; 0 (10–10); 93%
-Patients with suspected soft tissue sarcoma should ideally be reviewed by the sarcoma team and biopsied, within 2 weeks maximum (ideally 1 week)	10; 1 (9–10); 93%
**2.5. Interdisciplinary documentation:**	
-Preferably, all patients should be included in a soft tissue tumor database	10; 0 (10–10); 97%
-Standardized clinical record forms (CRF) should be used	10; 1 (9–10); 93%

## Discussion

The updated ESSR consensus guidelines aim to provide feasible best practice expert opinion pertaining to soft tissue tumor imaging. In comparison with the previous ESSR recommendations [11], the revised guidelines are updated to the current literature and re-structured. They provide minimal requirements and an optimized strategy in a systematic approach and contain relevant details.

The Delphi process was chosen as the panelists could perform their scoring anonymously and without the necessity to meet personally for rating [5]. However, additional face-to-face-meetings proved useful to clarify open questions regarding the procedure and to discuss concerns and re-phrasing of statements without consensus.

The extended expert panel included specialists from twelve European countries. The ESSR represents the European musculoskeletal radiologists [12]. Recruiting the panelists from the dedicated Musculoskeletal (MSK) tumor subcommittee of the ESSR allowed to form an adequate expert panel of active, representative, and leading specialists [13]. As group consensus (which reflects a considerably high level of agreement) could be reached in the majority of statements, and group agreement even in the remaining ones, this paper may help to provide feasible imaging algorithms taking into account different national infrastructure and approaches.

In this first part, the statements reflect the situation that any radiologist is confronted with in a patient with a newly suspected soft tissue tumor. Part I of our consensus therefore contains the imaging algorithm that we would recommend for primary diagnosis. It also contains detailed description of imaging methods for the tumor itself and the role of tumor reference centers and guidelines.

In the following paragraphs, we present a selection of the most clinically relevant statements with short discussion (the numbers correspond with Tables 1 and 2; the remaining comments are provided online).

### Primary diagnosis, local imaging

- 1.1.1. Regarding the past medical history, a standardized checklist, primarily filled out by the patient, and discussed with the radiologist, is considered advisable. The patient or the referring clinician should also be asked to provide previous imaging if available.
- 1.1.2. Information about clinical symptoms and clinical examination findings should be available for the radiologist.

The past medical history of the patient is considered important and has to be taken into account not only by the clinician, but also by the radiologist. A standardized checklist, primarily filled out by the patient, and discussed with the radiologist, is considered advisable [14]. The information that should be available for example includes recent trauma [15, 16], anticoagulation [17], and a history of previous surgery or of radiation therapy [18–20]. Of note, patients often report a recent trauma that they relate to the tumor, which, however, may be unrelated, and misleading [15, 16]. It is very important that the diagnostic process is not prolonged during the process of obtaining this information.

The patient or the referring clinician should also be asked if, where and when, previous imaging had been performed. The previous imaging studies and their radiological report should be provided to the assessing radiologist (if available) [11].

- 1.2.1*.* Ultrasound (US) is considered the appropriate initial triage imaging modality for a suspected soft tissue tumor, if accessible by US and small (< 5 cm). When US diagnosis is not typical for a diagnosis, refer to Magnetic Resonance Imaging (MRI) or even biopsy.
- *Caveat:* MRI should be performed prior to biopsy (if it will add to lesion characterization), not afterwards.
- 1.2.2. MRI is the imaging technique of choice for characterization and local staging of large (> 5 cm) musculoskeletal soft tissue masses and masses with indeterminate ultrasound features.
- Primary MRI should be considered instead of US if, there is a clinical suspicion of malignancy, if the mass is deep, rapidly enlarging, and if there is osseous or joint involvement.
- 1.2.3. Computed tomography (CT) can be considered instead of MRI for complex thoracic/ abdominal / other deep masses. CT should be performed in case of complex thoracic/ abdominal / other deep masses if MRI is unavailable or is contraindicated.

Ultrasound represents the initial triage imaging modality for accessible and small suspected soft tissue tumors [21–24]. Ultrasound is highly accurate for diagnosis of specific superficial lesions with typical ultrasound features [23, 25].

MRI is the modality of choice for the characterization and local staging of soft tissue tumors in most cases [26–28]. CT and MRI may have complementary roles, with the capability of CT to demonstrate intralesional mineralization patterns and potential bone involvement [29]. A deep soft tissue mass incidentally found at CT usually requires MRI examination. Tissue-specific evaluation and multiplanar capability of high-resolution MRI permit better tumor localization and characterization of pelvic/retroperitoneal masses [27, 30].

Suspicious or likely malignant tumors should undergo biopsy [11].

### Role of tumor centers and guidelines

- 2.1. Criteria for referral to a sarcoma treatment center include: Any patient with a tumor ≥ 5-cm, or with indeterminate or suspicious US/MRI findings, or with clinical suspicion of malignancy; Any patient with indeterminate MRI findings or those suspicious for malignancy.
- Teleradiologic second opinion from a tumor center is appropriate in patients with indeterminate or suspicious MRI findings. It should be offered to the local hospitals in all patients in whom soft tissue sarcoma is suspected.
- 2.2. Patients with suspicion of sarcoma should be referred to the tumor reference center ***before*** biopsy or surgery (minimal requirement).

In patients with large, indeterminate, or suspicious tumors, a tumor reference center should be contacted for referral or teleradiologic second opinion, to avoid delay in diagnosis or unplanned surgery (“whoops procedure”) [31–34], both of which can result in a potentially worse prognosis [35–38]. A second opinion MRI report from an expert center increases the overall accuracy in the diagnosis of soft tissue tumors, with fewer false-negative and false-positive diagnoses [39–41].

Biopsy of suspected appendicular soft tissue sarcoma should be performed by a tumor radiologist-specialist, using image guidance, to minimize adverse outcomes, and with minimal delay [42].

In case of unplanned surgery of sarcoma, the patients should immediately be referred to a sarcoma center for further evaluation and treatment, in order to avoid a potentially worse prognosis [35].

Local radiologists should implement guidelines for early imaging by ultrasound and MRI with a designated pathway. Adherence to those guidelines should on the one hand help prioritize onward referral for suspicious lesions [22], and on the other hand help reduce the volume of benign lesions referred [22, 43].

The imaging strategies that become necessary when the histologic diagnosis is already known will be covered in consecutive parts of our guidelines. This includes recommendations for whole-body staging in the primary diagnosis, for therapy control, and for follow-up imaging, as well as special aspects and pitfalls.

If these guidelines lead to more standardized examinations, the resulting data may be better suited for multicenter studies, with an improved possibility to collect and analyze comparable large data volumes. Thus, these guidelines may help to develop more individualized imaging protocols for the diagnosis of soft tissue tumors in the future.

### Limitations

Our consensus has got several limitations: The panelists came from European countries only. However, access to MRI is limited in many other parts of the world. In those areas, US and—if accessible—CT have to replace MRI. MRI contrast agents may be too expensive. Our guidelines take those points into account only to a certain extent. Tumor reference centers may be too distant, and teleradiologic consultation may not be available. In less-developed countries, only some parts of this consensus will be applicable at the moment. It is envisaged that these guidelines will however provide added impetus to health care professionals in these countries to canvass and optimize resources for better patient outcomes. The Delphi method itself has also got some limitations. Firstly, it is dependent on the expertise of the panelists. This was mostly overcome by including only ESSR-approved tumor specialists. Secondly, the possibility for open discussion is limited. On the other hand, this allows for distribution of critical remarks anonymously. Thus, the Delphi method had the advantage not to be biased by dominant participants. Thirdly, the process was time-consuming. This is a disadvantage that has been described for guidelines that contain multiple statements, such as our consensus [5]. As the Delphi process requires commitment to take part in several questionnaire rounds, we aimed to provide sufficient time for the participants to answer. Finally, it should be emphasized that these guidelines reflect the current knowledge and will require further updates in the future.

## Conclusion

The updated ESSR guidelines for imaging of soft tissue tumors in adults aim to provide best practice expert consensus for standardized imaging and are intended to support radiologists in their decision-making. Standardization may improve the comparability of serial examinations in the individual patient and may also provide databases for multicenter studies and large data analysis for individualized strategies.

### Supplementary information

Below is the link to the electronic supplementary material.

Supplementary file1 (PDF 511 KB)

## Abbreviations

AJCC: American Joint Committee on Cancer
CT: Computed tomography
ESSR: European Society of Musculoskeletal Radiology
MRI: Magnetic resonance imaging
MSK: Musculoskeletal
PMH: Past medical history
US: Ultrasound
WHO: World Health Organization

## Declarations

### Guarantor

The scientific guarantor of this publication is Univ.-Prof. Priv.-Doz. Dr. Iris-Melanie Noebauer-Huhmann.

### Statistics and biometry

No complex statistical methods were necessary for this paper.

### Study subjects or cohorts overlap

Study subjects or cohorts have not been previously reported.

### Methodology

*• *multicenter study
