# Supplementary material for: Soft tissue tumor imaging in adults: European Society of Musculoskeletal Radiology-Guidelines 2023—overview, and primary local imaging: how and where?
Source: Eur Radiol. 2023 Dec 7;34(7):4427–37. doi: 10.1007/s00330-023-10425-5 (PMC11213759; doi:10.1007/s00330-023-10425-5)
Supplement: Supplementary file 1 — Supplementary file1 (PDF 511 KB) [file 330_2023_10425_MOESM1_ESM.pdf]

Soft tissue tumor imaging in adults: European Society of Musculoskeletal Radiology - Guidelines 2023. Overview, and primary local imaging: How & where?

Electronic Supplementary Material

Statements and comments:

Section 1. Primary diagnosis, local imaging

1.1 History and Physical Examination ("H & P"):

1.1.1. Past medical history (PMH)

The following information should be available for the radiologist:

- When did the patient first notice the lesion?
- Does it change in size? Is it growing, and how fast?
- Has there been a recent trauma? Is the patient anticoagulated?
- Is there any oncologic history of the patient?
- Is there a family history of tumors or syndromes?
- Is there history of previous surgery or of radiation therapy?

A standardized checklist, primarily filled out by the patient, and discussed with the radiologist, is considered advisable. The patient or the referring clinician should also be asked to provide previous imaging if available.

Comments:

The past medical history of the patient is considered important and has to be taken into account not only by the clinician, but also by the radiologist. The following information regarding the PMH should be available for the radiologist:

(a) When did the patient first notice the lesion? (b) Is it growing, and how fast? Does it change in size (e.g., dependent on position, exercise, or muscle contraction/relaxation)? [1]

(c) Has there been a recent trauma? [2] Caveat: many patients report trauma that may be unrelated and misleading [3]. (d) Is the patient anticoagulated? [4] (e) Is there any oncologic history (malignancy, chemotherapy, or radiotherapy)? (f) Is there a family history of cancer, soft tissue tumors or syndroms [5] (e.g., Neurofibromatosis [6], Gardner syndrome [7], Li-Fraumeni syndrome [8], Retinoblastoma [9], Rothmund-Thomson syndrome [10], Werner syndrome [11], Gorlin syndrome [12], Tuberous sclerosis [13])? (g) Is there history of previous surgery or of radiation therapy? [14-16]

A standardized checklist [17], primarily filled out by the patient, and discussed with the radiologist, is considered advisable.
The patient or the referring clinician should also be asked if, where and when previous imaging had been performed . The previous imaging studies and their radiological report should be provided to the assessing radiologist (if available).[18]

1.1.2. Clinical symptoms and palpation

The following information should be available for the radiologist:

- Is the lesion palpable, and if so, is it hard or soft?
- Is it movable against the skin and underlying tissue?
- Is the lesion painful? Tinel sign?
- Are there skin alterations or pathologic vessels?
- Single or multiple lesions?

Comments:
The consistency of sarcomas often appears firm, while lipomas appear soft and vascular malformations can be compressed. [19] Fixed masses are often malignant, though locally aggressive lesions of intermediate dignity (such as desmoids) also may be fixed. [19]
The radiologist should know whether the lesion is painful (constant pain or pain on palpation), though indeterminate soft tissue tumors cannot be further characterized by this information.[20] Tumors of neural origin may be painful on palpation, and irritated nerves adjacent to tumors also may cause a tinel sign.[19]

Cutaneous sarcomas are rare, but there may be skin alterations or pathologic vessels adjacent to sarcoma.[3]
Typical examples for multiple lesions are vascular malformations [21] , tenosynovial giant cell tumors [22], desmoids [23], xanthomata [24, 25], Kaposi's sarcoma [26], and neurofibromas in Neurofibromatosis (NF) type 1 [6], schwannomas in NF2 [27] or in schwannomatosis. [28]

1.2 Imaging modalities and algorithm:

1.2.1. Role of Ultrasound.

- Ultrasound (US) is considered the **appropriate initial triage imaging modality** for a suspected soft tissue tumor, if accessible by US and small (<5cm). When US diagnosis is not typical for a diagnosis, refer to Magnetic Resonance Imaging (MRI) or even biopsy
  Caveat: MRI should be performed prior to biopsy (if it will add to lesion characterization), not afterwards

- Benign lesions that can be diagnosed on US include:
  - simple cyst, bursa, synovial/ganglion cyst (purely cystic well-defined lesion without any solid component, anechoic, with posterior acoustic enhancement and no internal vascularity);
  - superficial lipoma (homogeneous well defined, often encapsulated, and compressible with no clinical concern, clinically stable, < 10 cm and with documented stability on US (at least 6 months follow-up)),
  - foreign body granuloma with a compatible history,
  - superficial fibromatosis (e.g., palmar and plantar fibromatosis, infantile digit fibromatosis),
  - muscle hernia and
  - Morton neuroma.

- Benign lesions that can often be diagnosed on US include aneurysms and muscle tears. In any case of doubt, MRI should be performed.

- Small, superficial soft tissue masses that are likely to be benign, or which have been diagnosed with US (see above) but show interval growth should undergo biopsy (in lesions < 2-3 cm, excisional biopsy can be considered).

Comments:

In patients with a suspected soft tissue tumor, ultrasound (US) is considered the appropriate initial triage imaging modality if the tumor is accessible by US and <5cm in size [29-31]. The presence of a soft tissue tumor can be confirmed, but characterization is limited, as is local staging: invasion of juxta-articular structures, intra-articular extension and osseous involvement cannot be diagnosed reliably. US is readily available, "real time," radiation free, and cost effective but depends on the skill of the sonographer and may be challenging in obese patients. Reproducibility and reliability depend on consistent documentation.

Ultrasound is highly accurate for diagnosis of specific superficial lesions with typical ultrasound features [32] (see also section 1.4.1 on ultrasound report). Although US is excellent for the detection of even very small lesions, it may miss lesions in deep locations [32, 33]. Small areas of scar tissue can be misinterpreted as recurrence in patients who have had previous surgery. If a lesion is detected but not clearly benign, refer for biopsy or MRI [18]. Ultrasound should be used as primary guidance modality for biopsy in accessible areas [34]

US is able to provide a diagnosis of a benign soft tissue mass in the following lesions:

Simple cyst, bursa, synovial/ganglion cyst: purely cystic well-defined lesion without any solid component, anechoic, with posterior acoustic enhancement and no internal vascularity [34, 35]. Bursae [36] and ganglia [37] may show complex appearances but can also be diagnosed with US when they occur in typical sites and show otherwise compatible imaging features. Superficial lipomas are homogeneous, well defined, encapsulated and compressible, but show variable reflectivity; no significant internal vascularity on color Doppler, with no clinical concern and documented stability [38, 39] . Foreign body "granuloma" should be considered when there is a compatible history [40]. Superficial fibromatosis (e.g., palmar and plantar fibromatosis, infantile digital fibromatosis) [40-42]. Muscle hernia [43]. Morton neuroma can also be diagnosed by typical ultrasound features [44].

1.2.2. Role of Magnetic Resonance Imaging

- MRI is the imaging technique of choice for characterisation and local staging of musculoskeletal soft tissue masses with indeterminate ultrasound features and large tumors.
- Primary MRI should be considered instead of US if there is a clinical suspicion of malignancy, if the mass is deep, rapidly enlarging, and if there is osseous or joint involvement

---

Comments:

A soft tissue mass with indeterminate ultrasound features requires MR imaging, which is accepted as the imaging method of choice for diagnosis and local staging of musculoskeletal soft-tissue masses [45]. MRI should be performed for any large tumor with a chance of being malignant [46] .

Primary MRI should be performed as an initial imaging technique in the following indications [47]:

– Clinical suspicion of malignancy: suspicious clinical examination (fixed lump, large mass), deep mass, persistent swelling after trauma, rapidly enlarging lesion (except small superficial lesions), osseous involvement, joint involvement.

– In every case of abdominal/ pelvic/ retroperitoneal/ paravertebral mass lesion detected incidentally with other imaging modalities.

The following lesions can/may be reliably characterized by MRI:

- Anatomic variations, vascular malformation (+ high flow/low flow)
- ganglion cyst, Baker cyst, bursitis
- Lipoma, peripheral nerve sheath tumor (neurofibroma/schwannoma, apart from "ancient"schwannoma), TSGCT/pigmented villonodular synovitis (PVNS)
- hematoma, muscle tear, myositis ossificans, and aneurysm

Comments:

The following lesions can/may be reliably characterized by MRI:

- Anatomic variations, such as accessory muscles [48]

- Vascular malformation [49], either low flow (typically multiseptated masses of fluid-signal intensity with thin septations, signal voids, often containing fatty signal within or at the periphery, potential fluid-fluid levels due to stagnant flow), or high flow (typically with a cluster of irregular high flow vessels with one or more feeding arteries and one or more draining veins)

- Purely cystic lesions without any intralesional enhancement, such as Baker´s cysts, or a bursitis, intraneural ganglia at typical locations (i.e. peroneal nerve) [50, 51], or an abscess with rim enhancement and fluid content in the appropriate clinical setting.

- Lipoma [52, 53], in terms of an encapsulated or non-encapsulated lesion with fat signal intensity, potentially with thin (<2 mm) non enhancing septae. However, areas of higher signal intensity on T2 FS

images, due to inflammation, fibrosis, necrosis, hemorrhage, spindle cell lipomas, angiolipomas or brown fat in hibernomas may mimic well-differentiated liposarcomas. Superficial angiolipomas, (the rare) nerve lipomatosis, diffuse lipomatosis of the synovium (lipoma arborescens), (discrete) lipomas of the tendon sheath or joint [54, 55] and adiposis dolorosa subcutaneous lesions [56] can be confidently diagnosed on MR imaging.

- peripheral nerve sheath tumor (PNST), when there is typical relation to a nerve which appears tapered or normal [57] along with fusiform morphology, a bright T2 rim of the tumor, a target sign or fascicular sign (neurofibroma/schwannoma). However, definite characterization is only possible in cases of proven nonpainful neurofibromatosis (NF), when follow-up imaging is used to detect and monitor typical neurofibromas. Of note, so-called “ancient” schwannomas can often not be differentiated from malignant PNST [58]. A history of NF 1 is typical for large plexiform neurofibromata, while a history of NF2 for multiple schwannomas.

- Tenosynovial giant cell tumor (TSGCT)/pigmented villonodular synovitis (PVNS) [59], [60]. T2 hypointensity is characteristic for those tumors. Blooming effects are typically seen in the diffuse form of TSGCT due to their hemosiderin content, but may be missing in the rare localized intra-articular PVNS or TSGCT; in that subgroup, intense enhancement is often seen. A common subgroup of TSGT is related to a tendon sheath.

- Hematoma [61, 62], although the MRI appearances of chronic hematomas with peripheral nodular enhancement due to granulation tissue or large chronic expanding hematomas can be misleading

- Myositis ossificans (MO) [63], together with Projection radiography or CT; however in early stages, before the typical early peripheral mineralisation (eggshell-like in CT)develops, MO may masquerade sarcomas; alignment along the long axis of the muscle and extensive adjacent edema may be helpful [2]

- Muscle tear [64]. Rarely spontaneous (i.e. corticosteroid use, chronic renal failure) neglected ruptures of long muscles or tendons, such as Achilles, biceps or rectus femoris may present as pseudomasses [65].

- Aneurysm [66].

Other lesions that may be reliably characterized by MRI include lesions due to friction in certain anatomic locations, such as elastofibroma dorsi or plantar fibrous lesions under the metatarsal heads [67, 68], and also pseudotumors of peripheral nerves such as Morton neuromas, traumatic neuromas [69] and plantar / palmar fibromatoses [70].

1.2.3. Role of Projection Radiography

- There is limited role of radiographs in local staging of soft tissue sarcoma.
- However, radiography is a complementary modality for the identification and characterization of (a) intralesional mineralization patterns and (b) potential bone involvement of soft-tissue masses.

Comments:

Radiographs are cheap and easily available. These if performed can delineate fat within a lesion. Intralesional calcification and mineralization can be depicted [45, 71, 72]. This might be helpful in extraskeletal chondrosarcoma, synovial sarcoma, liposarcoma, chondroid lipoma and extra-skeletal mesenchymal chondrosarcoma. Characteristic calcifications can be pathognomonic in tumor mimics such as myositis ossificans, periostitis ossificans, or vascular malformation (phleboliths).

Soft tissue tumors adjacent to the bone may lead to pressure erosions or infiltrate with bone destruction (examples for pressure erosions are tenosynovial giant cell tumors, synovial chondromatosis or schwannomas, and examples for osseous infiltration are synovial sarcoma, extraskeletal chondrosarcoma, undifferentiated pleomorphic sarcoma, and liposarcoma) [71, 73].

The role of radiographs for local staging of soft tissue sarcoma is minimal [74].

1.2.4. Role of Computed Tomography

- For regions with a complex anatomy (e.g., axial skeleton, head/ neck, thoracic, and pelvic areas), CT is preferred over radiography.
- In cases where metallic structures cause unacceptable artifacts on MRI, even though modern metal artifact reduction techniques are applied, the use of CT with metal artifact reduction protocols may be useful.
- A deep soft tissue mass discovered incidentally during body CT requires further diagnosis. Depending on the lesion morphology, either MRI or immediate biopsy may be indicated.
- CT can be considered instead of MRI for complex thoracic/ abdominal / other deep masses. CT should be performed in case of complex thoracic/ abdominal / other deep masses if MRI is unavailable or contraindicated.
- Dual energy CT scan (DECT) can aid in metallic artefact reduction as well as in evaluation of soft tissue calcification.

Comments:

Primary CT should be considered instead of US when the lesion is intrathoracic or intra-abdominal (including pelvic and retroperitoneal lesions). CT after US should be considered where there are contraindications to MRI (such as pacemakers) or when MRI is not available.

In cases with metallic implants or other metal foreign bodies, which interfere with MRI imaging, CT can act as an alternative tool to delineate local tumor extension and its relation to the metallic hardware [75].

CT and MRI may have complementary roles, with the capability of CT to demonstrate intralesional mineralization patterns and potential bone involvement [76].

A deep soft tissue mass incidentally found at CT usually requires MRI examination. Tissue-specific evaluation and multiplanar capability of high-resolution MRI permit better tumor localization and characterization of pelvic / retroperitoneal masses [45, 46].

In conventional CT, differentiation between tissues are dependent on the atomic number of the materials involved, whereas in DECT, the difference in the attenuation is dependent on both the atomic number and electron density [77].
DECT can assess other aspects of musculoskeletal system including bone marrow edema and as a non-invasive alternative to synovial fluid aspiration in assessment of gout [78].
DECT can be effectively applied in reducing beam-hardening and metallic streak artefacts by using high-energy X-ray photons [77, 79]. DECT combined with post-processing by metal artefact reduction software can significantly enhance image quality [80].

1.2.5. Role of other techniques

- There is no role of bone scintigraphy in local staging of soft tissue sarcoma.
- There is no role of PET-MRI and MRS in routine local staging of soft tissue sarcoma

---

Comments:
There is no role of bone scintigraphy in local staging of soft tissue sarcoma [74, 81]. This has limited value even in distant staging as bone metastasis are rare in soft tissue sarcoma. Although literature is scarce, Technetium-99m SPECT might add to the specificity of radiologic imaging in lesions to prove bone formation (myositis ossificans) [82], and if the differential diagnosis includes specific rare primary soft tissue lesions (ossifying lipoma/osteolipoma, extraskeletal myxoid chondrosarcoma, chondroid lipoma, ossifying fibromyxoid tumor) [54].
IMT-SPECT might add to the detection of soft tissue sarcoma >5mm (a small study of 32 patients resulted in 100% sensitivity and 88% specificity) [83].
PET-MR and MR spectroscopy is used mainly for research purposes. These can enable evaluation of metabolic activity of soft tissue sarcoma and assessment of response post treatment and diagnosis of recurrence [84, 85].

1.3 Imaging technique:

1.3.1. Ultrasound technique

- The recommended frequency is at least 12 MHz. Lower frequencies can be used for detection of deeper lesions.
- Contrast enhanced Ultrasound (CEUS) can be considered by radiologists with special experience in CEUS for biopsy guidance in large lesions.
- Elastography is not considered necessary.
- Evidence regarding the application of ultrasound elastography for differentiating benign from malignant MSK soft tissue tumors is conflicting. There is no significant proof to recommend ultrasound elastography as a method for identification of MSK soft-tissue tumor malignancy.

Shearwave elastography is a feasible technique for evaluation of benign MSK soft tissue tumors, with insufficient proofs to be recommend as an imaging method for their differentiation.

Comments:

Minimal technical requirements (US probes, color-Doppler) are:

- Linear broadband array higher frequency probes, at least 12 MHz, frequency for deeper lesions not below 5–9 MHz.
- Extended field of view, harmonics and color-Doppler must be available.
- Complete visualization of the lesion and definition of the relation to bony structures is a minimum condition for local staging with US.
- Consider CEUS for detection of viable tissue in large lesions, especially for biopsy guidance [86].
- Not essential to have elastography and contrast [87-89].

Several recent studies showed potential usefulness of several shear-wave elastography measures in discrimination between benign and malignant MSK soft tissue tumors [90-92]. Other studies indicated low reproducibility [93] and low diagnostic performance [87, 94, 95], due to heterogeneity of soft tissue tumors [94, 95] and tumor surrounding tissue [95]. In superficial benign soft tissue tumor diagnosis the mean shear modulus was different between epidermoid cysts, ganglion cysts and superficial lipomas [96]. A study by Tavare et al [95] indicated that the shear wave velocity obtained by two dimensional shear wave elastography had significant diagnostic accuracy in discriminating MSK soft tissue lesions defined as probably or certainly benign by conventional ultrasound. It may provide an additional proof of the lesion benignity and help avoiding unnecessary biopsy if larger prospective studies confirm its utility.

1.3.2. MRI technique

– The recommended field strength of the MRI scanner for soft tissue tumors is at least 1.5 T. 3T may be useful and is optimal for advanced imaging such as spectroscopy.
– A cutaneous marker should be applied.
– The field of view should be as large as necessary to image the entire lesion, peritumoral edema, and a layer of adjacent normal tissue, and to image nonpalpable masses reliably.
– The voxel size should be as low as feasible to demonstrate relevant morphologic features and anatomic detail
– The size of the tumor should be measured in three dimensions.
– Axial sequences with high spatial resolution are important to define tumor margins, tissue and compartment involvement, and neurovascular, bone, and joint involvement.
– The recommended basic protocol includes combination of T1-weighted and a fluid-sensitive, fat-saturated (FS) sequence, both parallel to the long axis of the tumor.
– The use of Dixon technique for T2w and T1w sequences is advantageous, as a single Dixon based acquisition provides four contrasts, including images with and without fat suppression, and information about the fat content of a lesion (detection on Fat images and quantification on Fat Fraction maps).

- An axial T2-weighted sequence without fat saturation can provide further information about the tumor matrix.
- A diffusion weighted sequence (DWI) with calculation of the apparent diffusion coefficient (ADC) may also be useful.
- The diffusion-weighted sequence of the protocol should have at least two but optimally three b-values ranging from 50 s/mm2 to max. 800 or 1000 s/ mm^2.
- In MRI, intravenous gadolinium contrast administration with the use of dynamic contrast enhanced sequences (DCE) can help in the differentiation of benign versus malignant soft tissue tumors.
- DCE enables to detect viable intra-tumoral areas and to determine their vascularization patterns, and therefore assists in targeting tumor biopsy.
- Post Gd subtraction techniques are useful for ruling out areas which present an intrinsically high signal intensity on T1w images (such as melanin or methemoglobin).
- When applying appropriate metal artifact reduction MRI techniques, subtraction images of post- and pre- Gd T1w MR images are useful for assessing contrast enhancement next to metallic hardware, which may otherwise be obscured by failed spectral fat suppression.

Comments:

There are no studies that are directly comparing the diagnostic performance of 1.5 T and 3T magnets. Currently, literature data on the value of MRI in the diagnosis of Soft Tissue Tumors are mostly based on the use of a magnet strength of 1.5 T [97] . 1.5 T magnets are widely available and are known to provide good quality images and are useful for evaluation of soft tissue tumors in daily practice [98] . For more advanced imaging and multiparametric imaging, including Proton Nuclear MR Spectroscopy, 3 T magnets may add additional information [99-104] , but there is currently no evidence that this may alter clinical decision-making.

A cutaneous marker should be applied. Care should be taken not compress the mass by this marker [98]. A marker proximal and a second marker distal to the lesion is most appropriate to define the minimal FOV and to prevent local compression of the mass. The field of view should be as large as necessary to image the entire lesion, peritumoral edema, and a layer of adjacent normal tissue [105], and to image nonpalpable masses reliably (usually this aspect is especially important in coronal or sagittal sequences).The use of a large field-of-view (FOV) may be used for initial detection or for detection of multifocality. Multiplicity, satellite lesions, and abnormal proximal lymph nodes should be described on these large FOV. However use of a large FOV results in loss of spatial resolution; Therefore, a smaller FOV targeted to the lesion is recommended [98].

Axial sequences with high spatial resolution are important to define tumor margins, tissue and compartment involvement, and neurovascular, bone [106], and joint involvement [107]. The matrix should be optimized to achieve high in-plane spatial resolution, high signal-to-noise ratio, and a reasonable examination time.

The tumor should be measured in three dimensions (sequences should be performed in at least two planes). At least one sequence should include an external bony landmark for measuring and operation planning. Axial imaging is usually the primary imaging plane. The choice of the other imaging planes (either sagittal or coronal) depends on the location of the lesion. At least one T1-WI without fat-suppression is needed. T1-WI in more than one plane is useful if any fat is detected within the lesion [108].

Recommended basic and minimal imaging protocols include T1-weighted and a fluid-sensitive, fat-saturated (FS) sequence, both parallel to the long axis of the tumor. An axial T2-weighted and a T1-WI FS sequence may provide further information about the tumor matrix [108-110], and improve specificity. In T2-weighted sequences without fat saturation, hypointense areas may represent calcification or ossification, fibrous tissue, substance depositions (examples are hemosiderin, amyloid, or some gout tophi), or a combination of these [109]. Areas of the tumor which contain myxoid matrix are typically very hyperintense and may even appear fluid-like [111]. In those areas, diffusion weighted sequences (DWI) also show a high apparent diffusion coefficient (ADC) [112-114]. On the other hand, tumor stroma with densely packed cells (in tumors such as extraskeletal Ewing sarcoma) appears less hyperintense on T2w sequences, which, due to the image contrast, can be better differentiated without fat saturation, compared to fat saturated fluid sensitive sequences. In those tumors, the apparent diffusion coefficient (ADC) is typically markedly reduced [115] . Recommended b-values range from a minimal value of 50 s/mm2 (to minimize perfusion effects) to a maximal value of 800 s/mm2 (to avoid SNR degradation) [116, 117] or 1000 s/mm2 (to potentially provide even better conspicuity) [118].
The Dixon sequence, which was introduced in 1984 [119], is based on phase shifts created by fat–water resonance frequency differences, which allow to separate water from fat. A single acquisition results in four contrasts (“in phase”, “out of phase”, and, by addition or subtraction, also “water only” and “fat only”) [120]. A disadvantage is the need for longer scan times; however, with the development of mDixon (it uses asymmetrical echos, resulting in shorter acquisition time) the issue of time penalty can be addressed [121]. With technical advances, the technique became insensitive to B0 and B1 inhomogeneities (which posed problems especially in anatomical regions such as the head/neck region or the distal extremities, or areas near metallic objects), so that robust T1w, T2w and PD Dixon sequences can be assessed, including fast spin echo sequences [122] . Thus, e.g., the T2w sequence and the fluid sensitive fat suppressed sequence can be replaced by one T2w Dixon sequence, which also includes a reconstruction showing the fat content of a lesion. The fat content of lipomatous tumors has also been quantified by Dixon based sequences [123]. The fat suppression by Dixon T2 and Dixon T1-Gd FSE has also been tested specifically in MSK tumor imaging; it proved to be more homogeneous in areas of field inhomogeneity, compared with SPAIR [121]. A single T1w Dixon post Gadolinium can therefore replace the concurrent acquisition of T1w sequences with and without fat saturation [121]. If Dixon is not available, spectral fat suppression is preferred over inversion recovery unless metal artefact is present [108].
Although gradient echo imaging is not part of the routine imaging protocol, T2* imaging should be performed whenever the lesion is suspicious for containing hemosiderin [98].
Unless the lesion can be characterized as a definite benign lesion, such as a lipoma or a synovial cyst, we recommend using intravenous gadolinium-based contrast agents, if possible and feasible, at a routine dosage (usually, 0.1 mmol/kg body weight).
In patients with renal insufficiency, the European Society of Urogenital Radiology (ESUR) guidelines should be taken into account [124]. Post-contrast sequences should be performed in two planes (routinely, the T1-weighted pre-contrast sequence along the long axis is repeated, and a FS T1-weighted axial sequence is performed). Additional sequences may include dynamic contrast enhancement that can be helpful in characterizing the tumor (especially vascularization and matrix)

[102, 103, 106, 125, 126], and therefore assists in targeting tumor biopsy [127]. Pre-treatment dynamic MR studies are useful as a baseline for follow-up MR scans to monitor response to chemotherapy.
- Subtraction (the sequence after intravenous gadolinium contrast administration minus the identical sequence before) has been in use since the early days of MRI already [128]. It can help to differentiate enhancing areas from those which are intrinsically bright on T1 due to components with short T1 relaxation times (such as melanin, proteinaceous fluid, hemorrhagic areas with met-hemoglobin, or paramagnetic substances) [129, 130] (Gadolinium contrast subtraction has proved useful to reduce metal artifacts and thus allows a better visualization of tissue contrast enhancement next to metallic hardware [131].

1.3.3. Projection radiography technique

- Initial radiographic evaluation should be performed with at least two orthogonal views.

Comments:
Initial radiographic evaluation should be performed with two views at two orthogonal views [45, 71].

1.3.4. CT technique

- For the identification and characterization of intralesional mineralization patterns and potential bone involvement, CT without contrast agent application is sufficient.
- Iodinated contrast agents should be used in cases where CT serves for local staging instead of MRI.
- In case of metallic hardware, metal artefact reduction algorithms should be used.
- CT angiography (CTA) can be used for evaluation of the vascular encasement as well as in assessment of suspected tumoral thrombus of encased vessels.

Comments:
The use of soft tissue and bone windows, iodinated contrast, as well as multiplanar reconstruction capabilities of CT scan are important features contributing to accurate evaluation of local tumor extension and its relations to important surrounding structures [132]. However, for the identification and characterization of intralesional mineralization patterns and potential bone involvement, CT without contrast agent application is sufficient.
In cases with metallic implants or other metal foreign bodies, which interfere with MRI imaging, CT can act as a replacement method to delineate local tumor extension and its relation to the metallic hardware along with metal artefact reduction algorithms [75].
CT angiography (CTA) can be used in evaluating the vascular anatomy of a tumor as well as in the assessment of the degree of vascular relation/ compromise [76], though MRI is widely applied [133]. In cases when embolization is a treatment option; CT scan can be used for pre-operative planning.

1.4 Imaging reports should contain the following information:

1.4.1. Ultrasound reports

- Anatomical location: Relation to the fascia (superficial, deep), exact anatomical location including compartmental involvement, intra- or intermuscular location, and the relationship to/infiltration of vessels/nerves, and, if possible, joints and/or bone and crucial adjacent structures.
- Size in three dimensions (for the method, please see the section below on MRI).
- Morphology: borders/margins and shape (with estimation of growth pattern: infiltrative or expansive) and (if possible) presence of a capsule/pseudocapsule; cystic, solid (intralesional echo texture, vascularization (by color-Doppler based Giovagnorio classification), presence or absence of necrosis, bleeding, suspected tumor matrix mineralization)
- Concerns about tumor accessibility by US for a definitive diagnosis or the evaluation of local extension
- The fact that a lesion is indeterminate in US, with recommendation for subsequent imaging
- Change to previous examination/ tumor at the site of a previous excision

---

Comments:

For color-Doppler consider using the classification by Giovagnorio et al., in which avascular lesions are classified as type 1, hypovascular lesions feeding from single pole are classified as type 2, hypervascular lesions with multiple peripheral vessels are classified as type 3, and hypervascular lesions with multiple peripheral and central feedings are classified as type 4 [88, 134].
Although neoangiogenesis is a well-known paradigm in malignant tumors, and type 3 and type 4 lesions are most often malignant, it should be kept in mind that benign vascular soft tissue tumors like hemangiomas and vascular malformations may also demonstrate increased vascularity [88].

1.4.2. MRI reports *(for details, please see **checklist** in figure S1)*:

- Location and 3 D size, MR morphology, shape, border, relation to fascia,
- Intra- extracompartmental, relation to adjacent structures (vessels, nerves, joints,…) and surrounding tissue alterations
- Distance to external landmark, satellites, multiplicity, locoregional lymph nodes, and other tissue alterations.
- The image quality should be addressed.
- Changes to previous images (if available) should be described.

Comments:

The parameters to be described in local MRI serve to provide an exact local staging to target biopsy and plan the surgery or serve as a basic study before neoadjuvant therapy. They include parameters for the Enneking staging system [135] and the AJCC staging system [136].

Findings to be described include the following parameters:
Lesion location and extension, containing the region of the tumor, the relation to the deep peripheral fascia, intra- extracompartmental extension where applicable, with mentioning the compartments involved, relation to adjacent structures (muscles (inter-/intramuscular), growth along fascia/tail sign vessels, nerves, joints and/or bone) and to an external landmark, as well as the 3 D size [133, 135-143]. Different methods for lesion size measurement have been described. In the revised RECIST criteria, assessment of the longest lesion diameter in the acquired (axial) imaging plane is recommended [144]. With technical advancement, and especially with the isotropic reconstruction, it is possible to perform 3D measurements (calculation of the volume on this base is justified only in the case of regular lesions of spherical or ellipsoidal shape) or even semiautomated or automated volumetry [145]. To date, standardization is difficult, as none of those methods seems to correlate with pathological response to neoadjuvant chemotherapy and survival [146] (please also see section 2 on whole body staging).
The current AJCC TNM classification [147, 148] requires the assessment of the longest tumor diameter, which is also correlated with the prognosis [149]
A feasible standardized method for the description of the lesion size could be the following method used for Ewing sarcomas [142]: Measurement of the longest lesion diameter (a), definition of the perpendicular cross section, and measure the two largest perpendicular diameters (b,c) in that plane [142].
The MR morphology (matrix signal intensity (+ presence of fat, + of T2w hypointensity), homogeneity (esp. heterogeneity of >50% of the tumor volume on fluid-sensitive fat suppressed images, diffusion restriction (if available, with ADC) should be described. Moreover, the vascularity and enhancement (if available with enhancement pattern and, in inhomogeneous tumors, closer description of enhancement distribution), the presence of necrotic or hemorrhagic areas, calcification (evaluation together with radiograph or CT; pattern, if possible) ; shape, lobularity, borders (e.g., low T1, T2 signal intensity, pseudocapsule and /or contrast enhancement of the rim) [58, 150] , zone of transition, and surrounding tissue alterations [58, 105, 109, 150-156] should be described.
Satellites, multiplicity, locoregional lymph nodes, and other tissue alterations should be mentioned [108].Moreover, changes to previous images [157] and the image quality (esp. next to metal) should be addressed.

1.4.3. Projection radiographs reports

- Characteristic calcification patterns, bone destruction, and soft tissue swelling
- If possible: Density, location, longest diameter
- Also important features of unsuspected differential diagnosis
- Concerns about superposition effects with, if indicated, recommendation for cross-sectional imaging by CT

Comments:
The report should contain the description of characteristic calcification patterns, bone destruction, soft tissue swelling andif possible, density, location, and longest diameter. Important features of

unsuspected differential diagnosis, and concerns about superposition effects with, if indicated, recommendation for cross-sectional imaging by CT [71].

1.4.4. CT reports

- Size/Extension: location, longest diameter, bone (cortical and bone marrow) involvement (destruction/invasion, pressure arrosion/remodelling, sclerosis)
- Retroperitoneal liposarcoma: asymmetry in volume and extension of retroperitoneal fat
- Morphology: Density/attenuation, patterns of mineralization (e.g., phleboliths, ossification, osteoblastic, chondroid, dystrophic,) and its organization (scattered, peripherally or centrally mature), degree and pattern of vascularity/ contrast enhancement, necrosis.
- Margin, diffuse surrounding alterations such as stranding and inflammation, free fluid, free air, subsequent alterations of thoracic/abdominal organs (obstruction of ducts, small bowel, …)

Comments:

The report should contain the description of (1) Size/ extension of the lesion: Its location, longest diameter, bone (cortical and bone marrow) involvement (destruction/infiltration, pressure arrosion/remodelling, sclerosis,…) [158] (2) Morphology: Density/ attenuation, patterns of mineralization (e.g., phleboliths, ossification, osteoblastic, chondroid, dystrophic,) [108] and its organization (scattered, peripherally or centrally mature) [108], the degree and pattern of vascularity/ contrast enhancement, necrosis. The report should also comment on the margin, diffuse surrounding alterations such as stranding and inflammation, free fluid, free air, subsequent alterations of thoracic/abdominal organs (obstruction of ducts, small bowel, …) [23, 108, 158]. Retroperitoneal liposarcoma may be underdiagnosed due to the difficulty in differentiating from surrounding fat. Subtle difference of fat volume and asymmetry should be looked for, to diagnose these cases [159]

1.4.5. PET/CT reports

CT features + SUV, also satellite lesions and lymph nodes should be described [160, 161].

Section 2. The role of tumor centers and guidelines

2.1. Criteria for referral to or contacting a sarcoma treatment center

---

- Criteria for referral to a sarcoma treatment center include: Any patient with a tumor ≥ 5-cm, or with indeterminate or suspicious US findings, or with clinical suspicion of malignancy.
- Any patient with indeterminate MRI findings or those suspicious for malignancy.
- Teleradiologic second opinion workup by a tumor center is appropriate in patients with indeterminate or suspicious MRI findings. It should be offered to the local hospitals in all patients in whom soft tissue sarcoma is suspected.

Comments:

Patients with indeterminate soft tissue tumors or suspicion of sarcoma should be referred to a sarcoma treatment center for a primary or a secondary opinion, to avoid delay in diagnosis or unplanned surgery ("whoops procedure") [162-165], both of which can result in a potentially worse prognosis [166-169]. Clinical suspicion of sarcoma should be raised in patients with a painless lump or a lump that increases in size or other alarm symptoms, such as deep, large, rapidly growing. Those patients should be referred urgently to a sarcoma unit [170, 171].
Any patient with a ≥ 5-cm superficial tumor or with a deep-seated tumor regardless of size should be referred to a sarcoma unit [163, 172].
In superficial lesions less than 5 cm in maximal diameter, suspicion of sarcoma should be raised at least if there is fascial edema, skin thickening, skin contact, fast peripheral enhancement, hemorrhage, or necrosis [173]. Of note, in the latest, 8th, edition of the Cancer Staging Manual of the American Joint Committee on Cancer (AJCC), the tumor depths (in relation to the superficial fascia) has been erased as a criterion for the T-staging of soft tissue sarcomas [148].

A second opinion MRI report from an expert center increases the overall accuracy in the diagnosis of soft tissue tumors, with fewer false-negative and false-positive diagnoses [163, 174-176].
Problem in attendance of specialists due to geographical location is an important barrier to effective functioning of multidisciplinary teams (MDTs) [177]. Switch to virtual MDTs in sarcoma care following the unprecedented COVID-19 pandemic proved to be a viable and effective alternative to conventional face-to-face MDTs [178].
Some patients need referral to a tumor center due to the complexity of management even in non-malignant lesions.

2.2. Examinations that should be performed in a tumor reference center

---

- The accuracy in tumor characterisation may be higher if the MRI is performed and evaluated in a dedicated tumor centre. Where this is not feasible, the MRI scan should be performed as per the technical recommendations of the local tumor center.
- Patients with suspicion of sarcoma should be referred to the tumor reference center ***before*** biopsy or surgery (minimal requirement).

Comments:

In patients with suspicion of sarcoma, the accuracy in soft tissue tumor characterization was found to be higher in a study that performed and evaluated MRI in a dedicated tumor center, than the overall accuracy that had been described in the literature [97, 176].
Biopsy of suspected appendicular soft tissue sarcoma should be performed by a tumor radiologist-specialist, using image guidance, to minimize adverse outcomes, and with minimal delay [179].

In case of unplanned surgery of sarcoma, the patients should immediately be referred to a sarcoma center for further evaluation and treatment, in order to avoid a potentially worse prognosis [166].

2.3. Role of guidelines

- The guidelines are intended to provide international standards; by publication, and through further promotion by national specialized radiologists, the guidelines will ensure standardisation of high-quality soft tissue tumor diagnostic imaging.
- Radiologists should follow the local tumor center guidelines.

Comments:

Local radiologists should implement guidelines for early imaging by ultrasound and MRI with a designated pathway. Adherence to those guidelines should on the one hand help prioritize onward referral for suspicious lesions [31], and on the other hand help reduce the volume of benign lesions referred [31, 180].

2.4. Interdisciplinary tumor team

- Soft tissue tumor board: A multidisciplinary soft tissue sarcoma team should at least include an (orthopedic) tumor surgeon, a musculoskeletal radiologist, a musculoskeletal pathologist, a medical oncologist, and a radiotherapist. Where necessary, other specialists should be invited.
- An instant discussion between orthopedic tumor surgeon and a musculoskeletal radiologist improves service efficiency and reduces the time to definitive diagnosis.
- Patients with suspected soft tissue sarcoma should ideally be reviewed by the sarcoma team and biopsied, within 2 weeks maximum (ideally 1 week).

Comments:

Soft tissue sarcomas should be managed by a multidisciplinary sarcoma team that includes an (orthopedic) tumor surgeon, musculoskeletal radiologist (including nuclear medicine), and musculoskeletal pathologist [181].

A radiotherapist and an oncologist should also attend, as adjuvant RT can reduce the risk of local recurrence [182], and neo-adjuvant chemotherapy may be preferred in some cases. [183]

Further members of the team would for instance include other specialized surgeons, specialist sarcoma nurses or physiotherapists.

At the start of the pathway, an instant meeting between the orthopedic tumor surgeon and a musculoskeletal radiologist (ideally at the same or next day, after the first presentation of the patient, to discuss which additional measures should be taken before the board meeting), improves service efficiency and reduces the time to definitive diagnosis (TTDD) [184].

Early accurate diagnosis and appropriate treatment are crucial for optimum outcome [185].

Patients with suspected soft tissue sarcoma should ideally be reviewed by the sarcoma team and biopsied, if necessary, within 2 weeks maximum [186, 187].

Suboptimal pre-referral investigations and organization at a local hospital can increase the diagnostic interval by at least 1 month for 50% of the patients. If investigations are to be performed before referral to a sarcoma center, they should be part of the fast track pathway in order to ensure timely diagnosis [169].

2.5. Interdisciplinary documentation

- Preferably, all patients should be included in a soft tissue tumor database.
- Standardized clinical record forms (CRF) should be used.

Comments:

National soft tissue tumor databases have been introduced in various European countries already (among them:[188-196]).

Internationally accepted forms for histology reporting have been introduced [197].

Figure S1. Standardized Checklist MR Report:

Lesion location and extension

Region within the body: ☐ right ☐ left

- ☐ upper extremity, (shoulder, upper arm, elbow, forearm, wrist, hand)……………………………….
- ☐ lower extremity (hip, thigh, knee, lower leg, ankle, foot,…)…………………………………………. .
- ☐ trunk general: superficial (thoracic and abdominal wall, axilla, inguinal, para-vertebral) or deep: intrathoracic (mediastinal/cardiac, pleural), pelvic ……………………………………………….
- ☐ retroperitoneal…………………………………………………………………………………………………...
- ☐ head/neck …………………………………………………………………………………………………...
- ☐ visceral……………………………………………………………………………………………………….

Relation to fascia (superficial, deep) ………………………………………………………………………………..

Exact anatomical location, and intra-/extra-compartmental with compartments where applicable ..
……...

Relationship to adjacent tissues: muscles (inter-/intramuscular), growth along fascia/tail sign, infiltration of vessels/nerves, joints, and/or bone ……………………………………………………………………..
………
…………………………………………………………………………………………………..

Size in three dimensions (longest lesion diameter, definition of the perpendicular cross section, with measurement of the two largest perpendicular diameters in that plane)…………………………..
…….

Distance to external landmark ……………………………………………………………………………………..

Lesion morphology:

Matrix: Solid portion or purely cystic, matrix signal intensity (+ presence of fat, + of T2w hypointensity), homogeneity (esp. heterogeneity of >50% of the tumor volume on fluid-sensitive fat suppressed images), diffusion restriction (if available, with ADC) ………………………………………….
…….
…….

Vascularity and enhancement (if available with enhancement pattern), in inhomogeneous tumors closer description of enhancement distribution, presence of necrotic or hemorrhagic areas,
…….

Calcification (evaluation together with radiograph or CT; pattern, if possible………………………………….

Shape, lobularity, borders (with signal intensity (e.g., low T1, T2 signal intensity pseudocapsule) and/or contrast enhancement of the rim), zone of transition………………………………………………….
…….

Perilesional edema, perilesional contrast enhancement, tail-like patterns, surrounding tissue alterations
……………………………………………………………………………………………………….

Multiplicity and satellite lesions, abnormal proximal lymph nodes ………………………………………………..
…….

Changes in comparison to previous examination/ tumor at the site of a previous excision.
…….
…….

Image quality and assessability of the region, especially next to metal.
…….

REFERENCES

1. *Schmidt VF, Masthoff M, Czihal M, et al. (2021) Imaging of peripheral vascular malformations - current concepts and future perspectives. Mol Cell Pediatr, **8**(1):19 DOI: 10.1186/s40348-021-00132-w.*
2. *Savvidou O, Papakonstantinou O, Lakiotaki E, Melissaridou D, Korkolopoulou P and Papagelopoulos PJ (2021) Post-traumatic myositis ossificans: a benign lesion that simulates malignant bone and soft tissue tumours. EFORT Open Rev, **6**(7):572-583 DOI: 10.1302/2058-5241.6.210002.*
3. *Goulding KA, Wilke BK, Kiernan HC, Houdek MT and Sherman CE (2022) Skeletal Sarcomas: Diagnosis, Treatment, and Follow-up from the Orthopedic Oncologist Perspective. Radiol Clin North Am, **60**(2):193-203 DOI: 10.1016/j.rcl.2021.11.001.*
4. *Dohan A, Darnige L, Sapoval M and Pellerin O (2015) Spontaneous soft tissue hematomas. Diagn Interv Imaging, **96**(7-8):789-96 DOI: 10.1016/j.diii.2015.03.014.*
5. American_Cancer_Society. *Risk Factors for Soft Tissue Sarcomas*. cancer.org | 1.800.227.2345; Available from: https://www.cancer.org/cancer/soft-tissue-sarcoma/causes-risks-prevention/risk-factors.html.
6. *Nobauer-Huhmann IM, Brodowicz T and Marosi C (2018) How should adult patients with neurofibromatosis 1 be managed? Neuro Oncol, **20**(6):721-722 DOI: 10.1093/neuonc/noy050.*
7. rarediseases.info.nih.gov. *Gardner syndrome*. Available from: https://rarediseases.info.nih.gov/diseases/6482/gardner-syndrome.
8. *Correa H (2016) Li-Fraumeni Syndrome. J Pediatr Genet, **5**(2):84-8 DOI: 10.1055/s-0036-1579759.*
9. *Kleinerman RA, Schonfeld SJ, Sigel BS, et al. (2019) Bone and Soft-Tissue Sarcoma Risk in Long-Term Survivors of Hereditary Retinoblastoma Treated With Radiation. J Clin Oncol, **37**(35):3436-3445 DOI: 10.1200/JCO.19.01096.*
10. Wang LL and Plon SE, *Rothmund-Thomson Syndrome*, in *GeneReviews((R))*, M.P. Adam, et al., Editors. 1993: Seattle (WA).
11. *David A, Vincent M, Arrigoni PP, et al. (2017) Radiographic presentation of musculoskeletal involvement in Werner syndrome (adult progeria). Diagn Interv Imaging, **98**(5):373-378 DOI: 10.1016/j.diii.2016.10.007.*
12. *Guerrini-Rousseau L, Smith MJ, Kratz CP, et al. (2021) Current recommendations for cancer surveillance in Gorlin syndrome: a report from the SIOPE host genome working group (SIOPE HGWG). Fam Cancer, **20**(4):317-325 DOI: 10.1007/s10689-021-00247-z.*
13. *Ahmet Engin Atay , Halit Akbas, Nafi Sakar, Semir Pasa, Seyhmus Ari and f NE (2013) Clinical manifestations of tuberous sclerosis complex. Eastern Journal of Medicine **18**:52-57.*
14. *Lagrange JL, Ramaioli A, Chateau MC, et al. (2000) Sarcoma after radiation therapy: retrospective multiinstitutional study of 80 histologically confirmed cases. Radiation Therapist and Pathologist Groups of the Federation Nationale des Centres de Lutte Contre le Cancer. Radiology, **216**(1):197-205 DOI: 10.1148/radiology.216.1.r00jl02197.*
15. *Mavrogenis AF, Pala E, Guerra G and Ruggieri P (2012) Post-radiation sarcomas. Clinical outcome of 52 Patients. J Surg Oncol, **105**(6):570-6 DOI: 10.1002/jso.22122.*
16. *Funovics PT, Vaselic S, Panotopoulos J, Kotz RI and Dominkus M (2010) The impact of re-excision of inadequately resected soft tissue sarcomas on surgical therapy, results, and prognosis: A single institution experience with 682 patients. J Surg Oncol, **102**(6):626-33 DOI: 10.1002/jso.21639.*
17. *Rosenzweig MQ, Gardner D and Griffith B (2014) The History and Physical in Cancer Care: A Primer for the Oncology Advanced Practitioner. J Adv Pract Oncol, **5**(4):262-8 DOI: 10.6004/jadpro.2014.5.4.3.*
18. *Noebauer-Huhmann IM, Weber MA, Lalam RK, et al. (2015) Soft Tissue Tumors in Adults: ESSR-Approved Guidelines for Diagnostic Imaging. Semin Musculoskelet Radiol, **19**(5):475-82 DOI: 10.1055/s-0035-1569251.*
19. *Wilke BK, Goulding KA, Sherman CE and Houdek MT (2022) Soft Tissue Tumors: Diagnosis, Treatment, and Follow-up from the Orthopedic Oncologist Perspective. Radiol Clin North Am, **60**(2):253-262 DOI: 10.1016/j.rcl.2021.11.005.*
20. *Mayerson JL, Scharschmidt TJ, Lewis VO and Morris CD (2014) Diagnosis and Management of Soft-tissue Masses. J Am Acad Orthop Surg, **22**(11):742-50 DOI: 10.5435/JAAOS-22-11-742.*
21. *International Society for the Study of Vascular Anomalies*. Available from: https://www.issva.org/.
22. *Gouin F and Noailles T (2017) Localized and diffuse forms of tenosynovial giant cell tumor (formerly giant cell tumor of the tendon sheath and pigmented villonodular synovitis). Orthop Traumatol Surg Res, **103**(1S):S91-S97 DOI: 10.1016/j.otsr.2016.11.002.*

23. *Murphey MD, Ruble CM, Tyszko SM, Zbojniewicz AM, Potter BK and Miettinen M (2009) From the archives of the AFIP: musculoskeletal fibromatoses: radiologic-pathologic correlation. Radiographics,* ***29****(7):2143-73 DOI: 10.1148/rg.297095138.*
24. *Dagistan E, Canan A, Kizildag B and Barut AY (2013) Multiple tendon xanthomas in patient with heterozygous familial hypercholesterolaemia: sonographic and MRI findings. BMJ Case Rep,* ***2013*** *DOI: 10.1136/bcr-2013-200755.*
25. *Schweitzer ME and Karasick D (2000) MR imaging of disorders of the Achilles tendon. AJR Am J Roentgenol,* ***175****(3):613-25 DOI: 10.2214/ajr.175.3.1750613.*
26. *Yazici S, Zorlu O, Bulbul Baskan E, Balaban Adim S, Aydogan K and Saricaoglu H (2018) Retrospective Analysis of 91 Kaposi's Sarcoma Cases: A Single-Center Experience and Review of the Literature. Dermatology,* ***234****(5-6):205-213 DOI: 10.1159/000492112.*
27. *Evans DGR, Sainio M and Baser ME (2000) Neurofibromatosis type 2. Journal of Medical Genetics,* ***37****(12):897-904 DOI: DOI 10.1136/jmg.37.12.897.*
28. *Koontz NA, Wiens AL, Agarwal A, Hingtgen CM, Emerson RE and Mosier KM (2013) Schwannomatosis: the overlooked neurofibromatosis? AJR Am J Roentgenol,* ***200****(6):W646-53 DOI: 10.2214/AJR.12.8577.*
29. *Lakkaraju A, Sinha R, Garikipati R, Edward S and Robinson P (2009) Ultrasound for initial evaluation and triage of clinically suspicious soft-tissue masses. Clin Radiol,* ***64****(6):615-21 DOI: 10.1016/j.crad.2009.01.012.*
30. *Ritchie DA (2009) Commentary on ultrasound for initial evaluation and triage of clinically suspicious soft-tissue masses. Clin Radiol,* ***64****(6):622-3 DOI: 10.1016/j.crad.2009.02.008.*
31. *Rowbotham E, Bhuva S, Gupta H and Robinson P (2012) Assessment of referrals into the soft tissue sarcoma service: evaluation of imaging early in the pathway process. Sarcoma,* ***2012****:781723 DOI: 10.1155/2012/781723.*
32. *Hung EHY, Griffith JF, Yip SWY, et al. (2020) Accuracy of ultrasound in the characterization of superficial soft tissue tumors: a prospective study. Skeletal Radiol,* ***49****(6):883-892 DOI: 10.1007/s00256-019-03365-z.*
33. *Griffith JF, Yip SWY, Hung EHY, et al. (2020) Accuracy of ultrasound in the characterisation of deep soft tissue masses: a prospective study. Eur Radiol,* ***30****(11):5894-5903 DOI: 10.1007/s00330-020-07002-5.*
34. *Aparisi Gomez MP, Errani C, Lalam R, et al. (2020) The Role of Ultrasound in the Diagnosis of Soft Tissue Tumors. Semin Musculoskelet Radiol,* ***24****(2):135-155 DOI: 10.1055/s-0039-3402060.*
35. *Klauser AS, Tagliafico A, Allen GM, et al. (2012) Clinical indications for musculoskeletal ultrasound: a Delphi-based consensus paper of the European Society of Musculoskeletal Radiology. Eur Radiol,* ***22****(5):1140-8 DOI: 10.1007/s00330-011-2356-3.*
36. *Ruangchaijatuporn T, Gaetke-Udager K, Jacobson JA, Yablon CM and Morag Y (2017) Ultrasound evaluation of bursae: anatomy and pathological appearances. Skeletal Radiol,* ***46****(4):445-462 DOI: 10.1007/s00256-017-2577-x.*
37. *Teefey SA, Dahiya N, Middleton WD, Gelberman RH and Boyer MI (2008) Ganglia of the hand and wrist: a sonographic analysis. AJR Am J Roentgenol,* ***191****(3):716-20 DOI: 10.2214/AJR.07.3438.*
38. *Inampudi P, Jacobson JA, Fessell DP, et al. (2004) Soft-tissue lipomas: accuracy of sonography in diagnosis with pathologic correlation. Radiology,* ***233****(3):763-7 DOI: 10.1148/radiol.2333031410.*
39. *Wagner JM, Lee KS, Rosas H and Kliewer MA (2013) Accuracy of sonographic diagnosis of superficial masses. J Ultrasound Med,* ***32****(8):1443-50 DOI: 10.7863/ultra.32.8.1443.*
40. *Horton LK, Jacobson JA, Powell A, Fessell DP and Hayes CW (2001) Sonography and radiography of soft-tissue foreign bodies. AJR Am J Roentgenol,* ***176****(5):1155-9 DOI: 10.2214/ajr.176.5.1761155.*
41. *Griffith JF, Wong TY, Wong SM, Wong MW and Metreweli C (2002) Sonography of plantar fibromatosis. AJR Am J Roentgenol,* ***179****(5):1167-72 DOI: 10.2214/ajr.179.5.1791167.*
42. *Vanhoenacker FM, Eyselbergs M, Van Hul E, Van Dyck P and De Schepper AM (2011) Pseudotumoural soft tissue lesions of the hand and wrist: a pictorial review. Insights Imaging,* ***2****(3):319-333 DOI: 10.1007/s13244-011-0076-5.*
43. *Beggs I (2003) Sonography of muscle hernias. AJR Am J Roentgenol,* ***180****(2):395-9 DOI: 10.2214/ajr.180.2.1800395.*
44. *Quinn TJ, Jacobson JA, Craig JG and van Holsbeeck MT (2000) Sonography of Morton's neuromas. AJR Am J Roentgenol,* ***174****(6):1723-8 DOI: 10.2214/ajr.174.6.1741723.*
45. *Kransdorf MJ and Murphey MD (2016) Imaging of Soft-Tissue Musculoskeletal Masses: Fundamental Concepts. Radiographics,* ***36****(6):1931-1948 DOI: 10.1148/rg.2016160084.*

46. *Shanbhogue AK, Fasih N, Macdonald DB, Sheikh AM, Menias CO and Prasad SR (2012) Uncommon primary pelvic retroperitoneal masses in adults: a pattern-based imaging approach. Radiographics,* ***32****(3):795-817 DOI: 10.1148/rg.323115020.*
47. Brys P, *Magnetic Resonance Imaging: Basic Concepts*, in *Imaging of Soft Tissue Tumors*, F.M. Vanhoenacker, P.M. Parizel, and J.L. Gielen, Editors. 2017, Springer International Publishing: Cham. p. 71-83.
48. *Sookur PA, Naraghi AM, Bleakney RR, Jalan R, Chan O and White LM (2008) Accessory muscles: anatomy, symptoms, and radiologic evaluation. Radiographics,* ***28****(2):481-99 DOI: 10.1148/rg.282075064.*
49. *Merrow AC, Gupta A, Patel MN and Adams DM (2016) 2014 Revised Classification of Vascular Lesions from the International Society for the Study of Vascular Anomalies: Radiologic-Pathologic Update. Radiographics,* ***36****(5):1494-516 DOI: 10.1148/rg.2016150197.*
50. *Beaman FD and Peterson JJ (2007) MR imaging of cysts, ganglia, and bursae about the knee. Magn Reson Imaging Clin N Am,* ***15****(1):39-52 DOI: 10.1016/j.mric.2007.02.001.*
51. *Kalaci A, Karazincir S and Yanat AN (2007) Long-standing Morel-Lavallee lesion of the thigh simulating a neoplasm. Clin Imaging,* ***31****(4):287-91 DOI: 10.1016/j.clinimag.2007.01.012.*
52. *Kransdorf MJ, Bancroft LW, Peterson JJ, Murphey MD, Foster WC and Temple HT (2002) Imaging of fatty tumors: distinction of lipoma and well-differentiated liposarcoma. Radiology,* ***224****(1):99-104 DOI: 10.1148/radiol.2241011113.*
53. *Drevelegas A, Pilavaki M and Chourmouzi D (2004) Lipomatous tumors of soft tissue: MR appearance with histological correlation. Eur J Radiol,* ***50****(3):257-67 DOI: 10.1016/j.ejrad.2004.01.022.*
54. *Murphey MD, Carroll JF, Flemming DJ, Pope TL, Gannon FH and Kransdorf MJ (2004) From the archives of the AFIP: benign musculoskeletal lipomatous lesions. Radiographics,* ***24****(5):1433-66 DOI: 10.1148/rg.245045120.*
55. *Gupta P, Potti TA, Wuertzer SD, Lenchik L and Pacholke DA (2016) Spectrum of Fat-containing Soft-Tissue Masses at MR Imaging: The Common, the Uncommon, the Characteristic, and the Sometimes Confusing. Radiographics,* ***36****(3):753-66 DOI: 10.1148/rg.2016150133.*
56. *Tins BJ, Matthews C, Haddaway M, et al. (2013) Adiposis dolorosa (Dercum's disease): MRI and ultrasound appearances. Clin Radiol,* ***68****(10):1047-53 DOI: 10.1016/j.crad.2013.05.004.*
57. *Pressney I, Khoo M, Khan R, Abernethy P, Hargunani R and Saifuddin A (2021) Morphology of the entering and exiting nerve as a differentiating feature of benign from malignant peripheral nerve sheath tumours of the brachial plexus. Skeletal Radiol,* ***50****(8):1557-1565 DOI: 10.1007/s00256-020-03689-1.*
58. *Zhang Z, Deng L, Ding L and Meng Q (2015) MR imaging differentiation of malignant soft tissue tumors from peripheral schwannomas with large size and heterogeneous signal intensity. Eur J Radiol,* ***84****(5):940-6 DOI: 10.1016/j.ejrad.2015.02.003.*
59. *Sheldon PJ, Forrester DM and Learch TJ (2005) Imaging of intraarticular masses. Radiographics,* ***25****(1):105-19 DOI: 10.1148/rg.251045050.*
60. *Garner HW and Bestic JM (2013) Benign synovial tumors and proliferative processes. Semin Musculoskelet Radiol,* ***17****(2):177-8 DOI: 10.1055/s-0033-1343095.*
61. *Wu JS and Hochman MG (2009) Soft-tissue tumors and tumorlike lesions: a systematic imaging approach. Radiology,* ***253****(2):297-316 DOI: 10.1148/radiol.2532081199.*
62. *Bush CH (2000) The magnetic resonance imaging of musculoskeletal hemorrhage. Skeletal Radiol,* ***29****(1):1-9 DOI: 10.1007/s002560050001.*
63. *Parikh J, Hyare H and Saifuddin A (2002) The imaging features of post-traumatic myositis ossificans, with emphasis on MRI. Clin Radiol,* ***57****(12):1058-66 DOI: 10.1053/crad.2002.1120.*
64. *Guermazi A, Roemer FW, Robinson P, Tol JL, Regatte RR and Crema MD (2017) Imaging of Muscle Injuries in Sports Medicine: Sports Imaging Series. Radiology,* ***282****(3):646-663 DOI: 10.1148/radiol.2017160267.*
65. *Hemachandran N, Goyal A, Kandasamy D, Gamanagatti S, Srivastava DN and Ansari MT (2023) Myotendinous pseudomasses: an imaging review. Acta Radiol,* ***64****(1):172-186 DOI: 10.1177/02841851211061446.*
66. *Papp DF, Khanna AJ, McCarthy EF, Carrino JA, Farber AJ and Frassica FJ (2007) Magnetic resonance imaging of soft-tissue tumors: determinate and indeterminate lesions. J Bone Joint Surg Am,* ***89 Suppl 3****:103-15 DOI: 10.2106/JBJS.G.00711.*
67. *Studler U, Mengiardi B, Bode B, et al. (2008) Fibrosis and adventitious bursae in plantar fat pad of forefoot: MR imaging findings in asymptomatic volunteers and MR imaging-histologic comparison. Radiology,* ***246****(3):863-70 DOI: 10.1148/radiol.2463070196.*
68. *Bancroft LW, Pettis C and Wasyliw C (2013) Imaging of benign soft tissue tumors. Semin Musculoskelet Radiol,* ***17****(2):156-67 DOI: 10.1055/s-0033-1343071.*

69. *Tagliafico AS, Isaac A, Bignotti B, Rossi F, Zaottini F and Martinoli C (2019) Nerve Tumors: What the MSK Radiologist Should Know. Semin Musculoskelet Radiol, **23**(1):76-84 DOI: 10.1055/s-0038-1676290.*
70. *Dinauer PA, Brixey CJ, Moncur JT, Fanburg-Smith JC and Murphey MD (2007) Pathologic and MR imaging features of benign fibrous soft-tissue tumors in adults. Radiographics, **27**(1):173-87 DOI: 10.1148/rg.271065065.*
71. *Gartner L, Pearce CJ and Saifuddin A (2009) The role of the plain radiograph in the characterisation of soft tissue tumours. Skeletal Radiol, **38**(6):549-58 DOI: 10.1007/s00256-008-0513-9.*
72. *Kwee RM and Kwee TC (2019) Calcified or ossified benign soft tissue lesions that may simulate malignancy. Skeletal Radiol, **48**(12):1875-1890 DOI: 10.1007/s00256-019-03272-3.*
73. *Merino-Rueda LR, Barrientos-Ruiz I, Bernabeu-Taboada D, et al. (2022) Radiological and histopathological assessment of bone infiltration in soft tissue sarcomas. Eur J Orthop Surg Traumatol, **32**(4):631-639 DOI: 10.1007/s00590-021-03018-9.*
74. *Patel DB and Matcuk GR, Jr. (2018) Imaging of soft tissue sarcomas. Chin Clin Oncol, **7**(4):35 DOI: 10.21037/cco.2018.07.06.*
75. *Katsura M, Sato J, Akahane M, Kunimatsu A and Abe O (2018) Current and Novel Techniques for Metal Artifact Reduction at CT: Practical Guide for Radiologists. Radiographics, **38**(2):450-461 DOI: 10.1148/rg.2018170102.*
76. *Verga L, Brach Del Prever EM, Linari A, et al. (2016) Accuracy and role of contrast-enhanced CT in diagnosis and surgical planning in 88 soft tissue tumours of extremities. Eur Radiol, **26**(7):2400-8 DOI: 10.1007/s00330-015-4047-y.*
77. *Hamid S, Nasir MU, So A, Andrews G, Nicolaou S and Qamar SR (2021) Clinical Applications of Dual-Energy CT. Korean J Radiol, **22**(6):970-982 DOI: 10.3348/kjr.2020.0996.*
78. *Wang CK, Tsai JM, Chuang MT, Wang MT, Huang KY and Lin RM (2013) Bone marrow edema in vertebral compression fractures: detection with dual-energy CT. Radiology, **269**(2):525-33 DOI: 10.1148/radiology.13122577.*
79. *Stolzmann P, Winklhofer S, Schwendener N, Alkadhi H, Thali MJ and Ruder TD (2013) Monoenergetic computed tomography reconstructions reduce beam hardening artifacts from dental restorations. Forensic Sci Med Pathol, **9**(3):327-32 DOI: 10.1007/s12024-013-9420-z.*
80. *Shinohara Y, Sakamoto M, Iwata N, et al. (2014) Usefulness of monochromatic imaging with metal artifact reduction software for computed tomography angiography after intracranial aneurysm coil embolization. Acta Radiol, **55**(8):1015-23 DOI: 10.1177/0284185113510492.*
81. *Jager PL, Hoekstra HJ, Leeuw J, van Der Graaf WT, de Vries EG and Piers D (2000) Routine bone scintigraphy in primary staging of soft tissue sarcoma; Is it worthwhile? Cancer, **89**(8):1726-31 DOI: 10.1002/1097-0142(20001015)89:8<1726::aid-cncr12>3.0.co;2-v.*
82. *Sanchez Aguilar M, Garcia Jimenez R and Borrego Dorado I (2018) Imaging in myositis ossificans: Bone scintigraphy and 18F-fluorodeoxyglucose positron emission tomography/computed tomography. Reumatol Clin (Engl Ed), **14**(5):309-310 DOI: 10.1016/j.reuma.2017.01.017.*
83. *Jager PL, Plaat BE, de Vries EG, et al. (2000) Imaging of soft-tissue tumors using L-3-[iodine-123]iodo-alpha-methyl-tyrosine single photon emission computed tomography: comparison with proliferative and mitotic activity, cellularity, and vascularity. Clin Cancer Res, **6**(6):2252-9.*
84. *Zhang X, Chen YL, Lim R, Huang C, Chebib IA and El Fakhri G (2016) Synergistic role of simultaneous PET/MRI-MRS in soft tissue sarcoma metabolism imaging. Magn Reson Imaging, **34**(3):276-9 DOI: 10.1016/j.mri.2015.10.027.*
85. *Schuler MK, Richter S, Beuthien-Baumann B, et al. (2013) PET/MRI Imaging in High-Risk Sarcoma: First Findings and Solving Clinical Problems. Case Rep Oncol Med, **2013**:793927 DOI: 10.1155/2013/793927.*
86. *De Marchi A, Brach del Prever EM, Linari A, et al. (2010) Accuracy of core-needle biopsy after contrast-enhanced ultrasound in soft-tissue tumours. Eur Radiol, **20**(11):2740-8 DOI: 10.1007/s00330-010-1847-y.*
87. *Pass B, Jafari M, Rowbotham E, Hensor EM, Gupta H and Robinson P (2017) Do quantitative and qualitative shear wave elastography have a role in evaluating musculoskeletal soft tissue masses? Eur Radiol, **27**(2):723-731 DOI: 10.1007/s00330-016-4427-y.*
88. *Ozturk M, Selcuk MB, Polat AV, Ozbalci AB and Baris YS (2020) The diagnostic value of ultrasound and shear wave elastography in the differentiation of benign and malignant soft tissue tumors. Skeletal Radiol, **49**(11):1795-1805 DOI: 10.1007/s00256-020-03492-y.*

89. *Wu M, Ren A, Xu D, Peng X, Ye X and Li A (2021) Diagnostic Performance of Elastography in Malignant Soft Tissue Tumors: A Systematic Review and Meta-analysis. Ultrasound Med Biol, **47**(4):855-868 DOI: 10.1016/j.ultrasmedbio.2020.12.017.*
90. *Li A, Peng XJ, Ma Q, Dong Y, Mao CL and Hu Y (2020) Diagnostic performance of conventional ultrasound and quantitative and qualitative real-time shear wave elastography in musculoskeletal soft tissue tumors. J Orthop Surg Res, **15**(1):103 DOI: 10.1186/s13018-020-01620-x.*
91. *Pass B, Johnson M, Hensor EM, Gupta H and Robinson P (2016) Sonoelastography of Musculoskeletal Soft Tissue Masses: A Pilot Study of Quantitative Evaluation. J Ultrasound Med, **35**(10):2209-16 DOI: 10.7863/ultra.15.11065.*
92. *Ohshika S, Saruga T, Ogawa T, Ono H and Ishibashi Y (2021) Distinction between benign and malignant soft tissue tumors based on an ultrasonographic evaluation of vascularity and elasticity. Oncol Lett, **21**(4):281 DOI: 10.3892/ol.2021.12542.*
93. *Nicholls J, Alfuraih AM, Hensor EMA and Robinson P (2020) Inter- and intra-reader reproducibility of shear wave elastography measurements for musculoskeletal soft tissue masses. Skeletal Radiol, **49**(5):779-786 DOI: 10.1007/s00256-019-03300-2.*
94. *Winn N, Baldwin J, Cassar-Pullicino V, et al. (2020) Characterization of soft tissue tumours with ultrasound, shear wave elastography and MRI. Skeletal Radiol, **49**(6):869-881 DOI: 10.1007/s00256-019-03363-1.*
95. *Tavare AN, Alfuraih AM, Hensor EMA, Astrinakis E, Gupta H and Robinson P (2019) Shear-Wave Elastography of Benign versus Malignant Musculoskeletal Soft-Tissue Masses: Comparison with Conventional US and MRI. Radiology, **290**(2):410-417 DOI: 10.1148/radiol.2018180950.*
96. *Yeoh HJ, Kim TY and Ryu JA (2019) The feasibility of shear wave elastography for diagnosing superficial benign soft tissue masses. Ultrasonography, **38**(1):37-43 DOI: 10.14366/usg.17059.*
97. *Gielen JL, De Schepper AM, Vanhoenacker F, et al. (2004) Accuracy of MRI in characterization of soft tissue tumors and tumor-like lesions. A prospective study in 548 patients. Eur Radiol, **14**(12):2320-30 DOI: 10.1007/s00330-004-2431-0.*
98. Brys P, *Imaging of Soft Tissue Tumors*, in *Imaging of Soft Tissue Tumors, 4th edition*, F.M. Vanhoenacker, Paul M. Parizel, and J.L. Gielen, Editors. 2017, SPRINGER. p. 71-83.
99. *Fayad LM, Barker PB and Bluemke DA (2007) Molecular characterization of musculoskeletal tumors by proton MR spectroscopy. Semin Musculoskelet Radiol, **11**(3):240-5 DOI: 10.1055/s-2008-1038313.*
100. *Subhawong TK, Wang X, Durand DJ, et al. (2012) Proton MR spectroscopy in metabolic assessment of musculoskeletal lesions. AJR Am J Roentgenol, **198**(1):162-72 DOI: 10.2214/AJR.11.6505.*
101. *Lee CW, Lee JH, Kim DH, et al. (2010) Proton magnetic resonance spectroscopy of musculoskeletal lesions at 3 T with metabolite quantification. Clin Imaging, **34**(1):47-52 DOI: 10.1016/j.clinimag.2009.03.013.*
102. *Lee SK, Jee WH, Jung CK and Chung YG (2020) Multiparametric quantitative analysis of tumor perfusion and diffusion with 3T MRI: differentiation between benign and malignant soft tissue tumors. Br J Radiol, **93**(1115):20191035 DOI: 10.1259/bjr.20191035.*
103. *Dodin G, Salleron J, Jendoubi S, et al. (2021) Added-value of advanced magnetic resonance imaging to conventional morphologic analysis for the differentiation between benign and malignant non-fatty soft-tissue tumors. Eur Radiol, **31**(3):1536-1547 DOI: 10.1007/s00330-020-07190-0.*
104. Koenraad L. Verstraete, J. C. Dutoit, J. L. Drapé and Bloem JL, *Magnetic Resonance Imaging: Advanced Imaging Techniques*, in *Imaging of Soft Tissue Tumors, 4th edition*, F.M. Vanhoenacker, Paul M. Parizel, and J.L. Gielen, Editors. 2017, SPRINGER. p. 85-113.
105. *White LM, Wunder JS, Bell RS, et al. (2005) Histologic assessment of peritumoral edema in soft tissue sarcoma. Int J Radiat Oncol Biol Phys, **61**(5):1439-45 DOI: 10.1016/j.ijrobp.2004.08.036.*
106. *Choi YJ, Lee IS, Song YS, Kim JI, Choi KU and Song JW (2019) Diagnostic performance of diffusion-weighted (DWI) and dynamic contrast-enhanced (DCE) MRI for the differentiation of benign from malignant soft-tissue tumors. J Magn Reson Imaging, **50**(3):798-809 DOI: 10.1002/jmri.26607.*
107. Wörtler K, *Staging*, in *Imaging of Soft Tissue Tumors, 4th edition*, F.M. Vanhoenacker, Paul M. Parizel, and J.L. Gielen, Editors. 2017, SPRINGER. p. 145-159.
108. *Manaster BJ (2013) Soft-tissue masses: optimal imaging protocol and reporting. AJR Am J Roentgenol, **201**(3):505-14 DOI: 10.2214/AJR.13.10660.*
109. *Papakonstantinou O, Isaac A, Dalili D and Noebauer-Huhmann IM (2019) T2-weighted Hypointense Tumors and Tumor-like Lesions. Semin Musculoskelet Radiol, **23**(1):58-75 DOI: 10.1055/s-0038-1676126.*

110. *Gielen JL, De Schepper AM, Parizel PM, Wang XL and Vanhoenacker F (2003) Additional value of magnetic resonance with spin echo T1-weighted imaging with fat suppression in characterization of soft tissue tumors. J Comput Assist Tomogr, **27**(3):434-41 DOI: 10.1097/00004728-200305000-00024.*
111. *Baheti AD, Tirumani SH, Rosenthal MH, et al. (2015) Myxoid soft-tissue neoplasms: comprehensive update of the taxonomy and MRI features. AJR Am J Roentgenol, **204**(2):374-85 DOI: 10.2214/AJR.14.12888.*
112. *van Rijswijk CS, Kunz P, Hogendoorn PC, Taminiau AH, Doornbos J and Bloem JL (2002) Diffusion-weighted MRI in the characterization of soft-tissue tumors. J Magn Reson Imaging, **15**(3):302-7 DOI: 10.1002/jmri.10061.*
113. *Maeda M, Matsumine A, Kato H, et al. (2007) Soft-tissue tumors evaluated by line-scan diffusion-weighted imaging: influence of myxoid matrix on the apparent diffusion coefficient. J Magn Reson Imaging, **25**(6):1199-204 DOI: 10.1002/jmri.20931.*
114. *Subhawong TK, Jacobs MA and Fayad LM (2014) Diffusion-weighted MR imaging for characterizing musculoskeletal lesions. Radiographics, **34**(5):1163-77 DOI: 10.1148/rg.345140190.*
115. *Weber MA, Papakonstantinou O, Nikodinovska VV and Vanhoenacker FM (2019) Ewing's Sarcoma and Primary Osseous Lymphoma: Spectrum of Imaging Appearances. Semin Musculoskelet Radiol, **23**(1):36-57 DOI: 10.1055/s-0038-1676125.*
116. *Subhawong TK, Jacobs MA and Fayad LM (2014) Insights into quantitative diffusion-weighted MRI for musculoskeletal tumor imaging. AJR Am J Roentgenol, **203**(3):560-72 DOI: 10.2214/AJR.13.12165.*
117. *Guirguis M, Sharan G, Wang J and Chhabra A (2022) Diffusion-weighted MR imaging of musculoskeletal tissues: incremental role over conventional MR imaging in bone, soft tissue, and nerve lesions. BJR Open, **4**(1):20210077 DOI: 10.1259/bjro.20210077.*
118. *Tang L and Zhou XJ (2019) Diffusion MRI of cancer: From low to high b-values. J Magn Reson Imaging, **49**(1):23-40 DOI: 10.1002/jmri.26293.*
119. *Dixon WT (1984) Simple proton spectroscopic imaging. Radiology, **153**(1):189-94 DOI: 10.1148/radiology.153.1.6089263.*
120. *Bley TA, Wieben O, Francois CJ, Brittain JH and Reeder SB (2010) Fat and water magnetic resonance imaging. J Magn Reson Imaging, **31**(1):4-18 DOI: 10.1002/jmri.21895.*
121. *Huijgen WHF, van Rijswijk CSP and Bloem JL (2019) Is fat suppression in T1 and T2 FSE with mDixon superior to the frequency selection-based SPAIR technique in musculoskeletal tumor imaging? Skeletal Radiol, **48**(12):1905-1914 DOI: 10.1007/s00256-019-03227-8.*
122. *Low RN, Austin MJ and Ma J (2011) Fast spin-echo triple echo dixon: Initial clinical experience with a novel pulse sequence for simultaneous fat-suppressed and nonfat-suppressed T2-weighted spine magnetic resonance imaging. J Magn Reson Imaging, **33**(2):390-400 DOI: 10.1002/jmri.22453.*
123. *Skorpil M, Ryden H, Berglund J, Brynolfsson P, Brosjo O and Tsagozis P (2019) Soft-tissue fat tumours: differentiating malignant from benign using proton density fat fraction quantification MRI. Clin Radiol, **74**(7):534-538 DOI: 10.1016/j.crad.2019.01.011.*
124. [Internet]. *ESUR guidelines on contrast media*. Rentgenologiya i Radiologiya 2018; Available from: Available from: https://www.esur.org/fileadmin/content/2019/ESUR_Guidelines_10.0_Final_Version.pdf.
125. *Sujlana P, Skrok J and Fayad LM (2018) Review of dynamic contrast-enhanced MRI: Technical aspects and applications in the musculoskeletal system. J Magn Reson Imaging, **47**(4):875-890 DOI: 10.1002/jmri.25810.*
126. *Ahlawat S, Fritz J, Morris CD and Fayad LM (2019) Magnetic resonance imaging biomarkers in musculoskeletal soft tissue tumors: Review of conventional features and focus on nonmorphologic imaging. J Magn Reson Imaging, **50**(1):11-27 DOI: 10.1002/jmri.26659.*
127. *Noebauer-Huhmann IM, Amann G, Krssak M, et al. (2015) Use of diagnostic dynamic contrast-enhanced (DCE)-MRI for targeting of soft tissue tumour biopsies at 3T: preliminary results. Eur Radiol, **25**(7):2041-8 DOI: 10.1007/s00330-014-3576-0.*
128. *Hanna SL, Langston JW, Gronemeyer SA and Fletcher BD (1990) Subtraction technique for contrast-enhanced MR images of musculoskeletal tumors. Magn Reson Imaging, **8**(3):213-5 DOI: 10.1016/0730-725x(90)90091-f.*
129. *Downs RK, Bashir MH, Ng CK and Heidenreich JO (2013) Quantitative contrast ratio comparison between T1 (TSE at 1.5T, FLAIR at 3T), magnetization prepared rapid gradient echo and subtraction imaging at 1.5T and 3T. Quant Imaging Med Surg, **3**(3):141-6 DOI: 10.3978/j.issn.2223-4292.2013.05.02.*
130. *Eid M and Abougabal A (2014) Subtraction images: A really helpful tool in non-vascular MRI. The Egyptian Journal of Radiology and Nuclear Medicine, **45**(3):909-191 DOI: doi.org/10.1016/j.ejrnm.2014.04.013.*

131. *Muller GM, Mansson S, Muller MF, et al. (2014) MR imaging with metal artifact-reducing sequences and gadolinium contrast agent in a case-control study of periprosthetic abnormalities in patients with metal-on-metal hip prostheses. Skeletal Radiol,* ***43****(8):1101-12 DOI: 10.1007/s00256-014-1893-7.*
132. *Yamamoto T, Kurosaka M, Soejima T and Fujii M (2001) Contrast-enhanced three-dimensional helical CT for soft tissue tumors in the extremities. Skeletal Radiol,* ***30****(7):384-7 DOI: 10.1007/s002560100353.*
133. *Holzapfel K, Regler J, Baum T, et al. (2015) Local Staging of Soft-Tissue Sarcoma: Emphasis on Assessment of Neurovascular Encasement-Value of MR Imaging in 174 Confirmed Cases. Radiology,* ***275****(2):501-9 DOI: 10.1148/radiol.14140510.*
134. *Giovagnorio F, Andreoli C and De Cicco ML (1999) Color Doppler sonography of focal lesions of the skin and subcutaneous tissue. J Ultrasound Med,* ***18****(2):89-93 DOI: 10.7863/jum.1999.18.2.89.*
135. *Enneking WF (1986) A system of staging musculoskeletal neoplasms. Clin Orthop Relat Res, (204):9-24.*
136. in *AJCC Cancer Staging Manual*, Mahul B. Amin, et al., Editors. 2017, SPRINGER.
137. *Datir A, James SL, Ali K, Lee J, Ahmad M and Saifuddin A (2008) MRI of soft-tissue masses: the relationship between lesion size, depth, and diagnosis. Clin Radiol,* ***63****(4):373-8; discussion 379-80 DOI: 10.1016/j.crad.2007.08.016.*
138. *Morel M, Taieb S, Penel N, et al. (2011) Imaging of the most frequent superficial soft-tissue sarcomas. Skeletal Radiol,* ***40****(3):271-84 DOI: 10.1007/s00256-009-0855-y.*
139. *Yoo HJ, Hong SH, Kang Y, et al. (2014) MR imaging of myxofibrosarcoma and undifferentiated sarcoma with emphasis on tail sign; diagnostic and prognostic value. Eur Radiol,* ***24****(8):1749-57 DOI: 10.1007/s00330-014-3181-2.*
140. *Toomayan GA, Robertson F and Major NM (2005) Lower extremity compartmental anatomy: clinical relevance to radiologists. Skeletal Radiol,* ***34****(6):307-13 DOI: 10.1007/s00256-005-0910-2.*
141. *Toomayan GA, Robertson F, Major NM and Brigman BE (2006) Upper extremity compartmental anatomy: clinical relevance to radiologists. Skeletal Radiol,* ***35****(4):195-201 DOI: 10.1007/s00256-005-0063-3.*
142. *Aghighi M, Boe J, Rosenberg J, et al. (2016) Three-dimensional Radiologic Assessment of Chemotherapy Response in Ewing Sarcoma Can Be Used to Predict Clinical Outcome. Radiology,* ***280****(3):905-15 DOI: 10.1148/radiol.2016151301.*
143. Kransdorf MJ and Murphey MD, *Imaging of Soft Tissue Tumors, Third Edition*. 2013, Lippincott Williams & Wilkins.
144. *Eisenhauer EA, Therasse P, Bogaerts J, et al. (2009) New response evaluation criteria in solid tumours: revised RECIST guideline (version 1.1). Eur J Cancer,* ***45****(2):228-47 DOI: 10.1016/j.ejca.2008.10.026.*
145. *Gowin E, Jonczyk-Potoczna K, Sosnowska-Sienkiewicz P, Belen Larque A, Kurzawa P and Januszkiewicz-Lewandowska D (2021) Semi-Automatic Volumetric and Standard Three-Dimensional Measurements for Primary Tumor Evaluation and Response to Treatment Assessment in Pediatric Rhabdomyosarcoma. J Pers Med,* ***11****(8) DOI: 10.3390/jpm11080717.*
146. *Tanaka K, Ogawa G, Mizusawa J, et al. (2018) Prospective comparison of various radiological response criteria and pathological response to preoperative chemotherapy and survival in operable high-grade soft tissue sarcomas in the Japan Clinical Oncology Group study JCOG0304. World J Surg Oncol,* ***16****(1):162 DOI: 10.1186/s12957-018-1462-y.*
147. *Tanaka K and Ozaki T (2019) New TNM classification (AJCC eighth edition) of bone and soft tissue sarcomas: JCOG Bone and Soft Tissue Tumor Study Group. Jpn J Clin Oncol,* ***49****(2):103-107 DOI: 10.1093/jjco/hyy157.*
148. *Amin MB, Greene FL, Edge SB, et al. (2017) The Eighth Edition AJCC Cancer Staging Manual: Continuing to build a bridge from a population-based to a more "personalized" approach to cancer staging. CA Cancer J Clin,* ***67****(2):93-99 DOI: 10.3322/caac.21388.*
149. *Squires MH, Ethun CG, Donahue EE, et al. (2022) Extremity Soft Tissue Sarcoma: A Multi-Institutional Validation of Prognostic Nomograms. Ann Surg Oncol,* ***29****(5):3291-3301 DOI: 10.1245/s10434-021-11205-5.*
150. *Crombe A, Marcellin PJ, Buy X, et al. (2019) Soft-Tissue Sarcomas: Assessment of MRI Features Correlating with Histologic Grade and Patient Outcome. Radiology,* ***291****(3):710-721 DOI: 10.1148/radiol.2019181659.*
151. *van Rijswijk CS, Geirnaerdt MJ, Hogendoorn PC, et al. (2004) Soft-tissue tumors: value of static and dynamic gadopentetate dimeglumine-enhanced MR imaging in prediction of malignancy. Radiology,* ***233****(2):493-502 DOI: 10.1148/radiol.2332031110.*
152. *Teixeira PAG, Renaud A, Aubert S, et al. (2018) Perfusion MR imaging at 3-Tesla: Can it predict tumor grade and histologic necrosis rate of musculoskeletal sarcoma? Diagnostic and Interventional Imaging,* ***99****(7-8):473-481 DOI: 10.1016/j.diii.2018.02.005.*

153. *Verstraete KL and Lang P (2000) Bone and soft tissue tumors: the role of contrast agents for MR imaging. Eur J Radiol, **34**(3):229-46 DOI: 10.1016/s0720-048x(00)00202-3.*
154. *Lefkowitz RA, Landa J, Hwang S, et al. (2013) Myxofibrosarcoma: prevalence and diagnostic value of the "tail sign" on magnetic resonance imaging. Skeletal Radiology, **42**(6):809-818 DOI: 10.1007/s00256-012-1563-6.*
155. *Walker EA, Petscavage JM, Brian PL, Logie CI, Montini KM and Murphey MD (2012) Imaging features of superficial and deep fibromatoses in the adult population. Sarcoma, **2012**:215810 DOI: 10.1155/2012/215810.*
156. *Morii T, Tajima T, Honya K, Aoyagi T and Ichimura S (2018) Clinical significance of the tail-like pattern in soft-tissue sarcomas on magnetic resonance imaging. J Orthop Sci, **23**(6):1032-1037 DOI: 10.1016/j.jos.2018.06.010.*
157. *Noebauer-Huhmann IM, Chaudhary SR, Papakonstantinou O, et al. (2020) Soft Tissue Sarcoma Follow-up Imaging: Strategies to Distinguish Post-treatment Changes from Recurrence. Semin Musculoskelet Radiol, **24**(6):627-644 DOI: 10.1055/s-0040-1721464.*
158. *Subhawong TK, Fishman EK, Swart JE, Carrino JA, Attar S and Fayad LM (2010) Soft-tissue masses and masslike conditions: what does CT add to diagnosis and management? AJR Am J Roentgenol, **194**(6):1559-67 DOI: 10.2214/AJR.09.3736.*
159. *Kirane A and Crago AM (2016) The importance of surgical margins in retroperitoneal sarcoma. J Surg Oncol, **113**(3):270-6 DOI: 10.1002/jso.24135.*
160. *Macpherson RE, Pratap S, Tyrrell H, et al. (2018) Retrospective audit of 957 consecutive (18)F-FDG PET-CT scans compared to CT and MRI in 493 patients with different histological subtypes of bone and soft tissue sarcoma. Clin Sarcoma Res, **8**:9 DOI: 10.1186/s13569-018-0095-9.*
161. *Roberge D, Vakilian S, Alabed YZ, Turcotte RE, Freeman CR and Hickeson M (2012) FDG PET/CT in Initial Staging of Adult Soft-Tissue Sarcoma. Sarcoma, **2012**:960194 DOI: 10.1155/2012/960194.*
162. *Bianchi G, Sambri A, Cammelli S, et al. (2017) Impact of residual disease after "unplanned excision" of primary localized adult soft tissue sarcoma of the extremities: evaluation of 452 cases at a single Institution. Musculoskelet Surg, **101**(3):243-248 DOI: 10.1007/s12306-017-0475-y.*
163. *Lazarides AL, Kerr DL, Nussbaum DP, et al. (2019) Soft Tissue Sarcoma of the Extremities: What Is the Value of Treating at High-volume Centers? Clin Orthop Relat Res, **477**(4):718-727 DOI: 10.1097/01.blo.0000533623.60399.1b.*
164. *Wang L, Pretell-Mazzini J, Kerr DA, et al. (2018) MRI findings associated with microscopic residual tumor following unplanned excision of soft tissue sarcomas in the extremities. Skeletal Radiol, **47**(2):181-190 DOI: 10.1007/s00256-017-2762-y.*
165. *Tedesco NS and Henshaw RM (2016) Unplanned Resection of Sarcoma. J Am Acad Orthop Surg, **24**(3):150-9 DOI: 10.5435/JAAOS-D-15-00074.*
166. *Umer HM, Umer M, Qadir I, Abbasi N and Masood N (2013) Impact of unplanned excision on prognosis of patients with extremity soft tissue sarcoma. Sarcoma, **2013**:498604 DOI: 10.1155/2013/498604.*
167. *Abellan JF, Lamo de Espinosa JM, Duart J, et al. (2009) Nonreferral of possible soft tissue sarcomas in adults: a dangerous omission in policy. Sarcoma, **2009**:827912 DOI: 10.1155/2009/827912.*
168. *Traub F, Griffin AM, Wunder JS and Ferguson PC (2018) Influence of unplanned excisions on the outcomes of patients with stage III extremity soft-tissue sarcoma. Cancer, **124**(19):3868-3875 DOI: 10.1002/cncr.31648.*
169. *Dyrop HB, Vedsted P, Raedkjaer M, Safwat A and Keller J (2017) Imaging investigations before referral to a sarcoma center delay the final diagnosis of musculoskeletal sarcoma. Acta Orthop, **88**(2):211-216 DOI: 10.1080/17453674.2016.1278113.*
170. *George A and Grimer R (2012) Early symptoms of bone and soft tissue sarcomas: could they be diagnosed earlier? Ann R Coll Surg Engl, **94**(4):261-6 DOI: 10.1308/003588412X13171221590016.*
171. *Dyrop HB, Safwat A, Vedsted P, et al. (2016) Characteristics of 64 sarcoma patients referred to a sarcoma center after unplanned excision. J Surg Oncol, **113**(2):235-9 DOI: 10.1002/jso.24137.*
172. *Styring E, Billing V, Hartman L, et al. (2012) Simple guidelines for efficient referral of soft-tissue sarcomas: a population-based evaluation of adherence to guidelines and referral patterns. J Bone Joint Surg Am, **94**(14):1291-6 DOI: 10.2106/JBJS.K.01271.*
173. *Calleja M, Dimigen M and Saifuddin A (2012) MRI of superficial soft tissue masses: analysis of features useful in distinguishing between benign and malignant lesions. Skeletal Radiol, **41**(12):1517-24 DOI: 10.1007/s00256-012-1385-6.*

174. *Vanhoenacker FM, Van Looveren K, Trap K, et al. (2012) Grading and characterization of soft tissue tumors on magnetic resonance imaging: the value of an expert second opinion report. Insights Imaging, **3**(2):131-8 DOI: 10.1007/s13244-012-0151-6.*
175. *Bagaria SP, Neville M, Gray RJ, et al. (2018) The Volume-Outcome Relationship in Retroperitoneal Soft Tissue Sarcoma: Evidence of Improved Short- and Long-Term Outcomes at High-Volume Institutions. Sarcoma, **2018**:3056562 DOI: 10.1155/2018/3056562.*
176. *Rozenberg A, Kenneally BE, Abraham JA, et al. (2019) Second opinions in orthopedic oncology imaging: can fellowship training reduce clinically significant discrepancies? Skeletal Radiol, **48**(1):143-147 DOI: 10.1007/s00256-018-3024-3.*
177. *Fayet Y, Tetreau R, Honore C, et al. (2021) Determinants of the access to remote specialised services provided by national sarcoma reference centres. BMC Cancer, **21**(1):631 DOI: 10.1186/s12885-021-08393-4.*
178. *Rajasekaran RB, Whitwell D, Cosker TDA, Gibbons C and Carr A (2021) Will virtual multidisciplinary team meetings become the norm for musculoskeletal oncology care following the COVID-19 pandemic? - experience from a tertiary sarcoma centre. BMC Musculoskelet Disord, **22**(1):18 DOI: 10.1186/s12891-020-03925-8.*
179. *Elliott RS, Flint M and French G (2012) Refer prior to biopsy of suspected appendicular soft tissue sarcoma. N Z Med J, **125**(1366):12-9.*
180. *Pencavel TD, Strauss DC, Thomas GP, Thomas JM and Hayes AJ (2010) Does the two-week rule pathway improve the diagnosis of soft tissue sarcoma? A retrospective review of referral patterns and outcomes over five years in a regional sarcoma centre. Ann R Coll Surg Engl, **92**(5):417-21 DOI: 10.1308/003588410X12664192075972.*
181. *Misra A, Mistry N, Grimer R and Peart F (2009) The management of soft tissue sarcoma. J Plast Reconstr Aesthet Surg, **62**(2):161-74 DOI: 10.1016/j.bjps.2008.08.018.*
182. *Fujiwara T, Tsuda Y, Le Nail LR, et al. (2020) The role of radiotherapy in the treatment of superficial soft-tissue sarcomas. Bone Joint J, **102-B**(8):1088-1094 DOI: 10.1302/0301-620X.102B8.BJJ-2020-0043.R1.*
183. *NCCN Guidelines*. Soft tissue sacoma 2019; Available from: http://www.nccn.org.
184. *Hartley LJ, Evans S, Davies MA, Kelly S and Gregory JJ (2021) A Daily Diagnostic Multidisciplinary Meeting to Reduce Time to Definitive Diagnosis in the Context of Primary Bone and Soft Tissue Sarcoma. J Multidiscip Healthc, **14**:115-123 DOI: 10.2147/JMDH.S266014.*
185. *Nakamura T, Matsumine A, Matsubara T, Asanuma K, Uchida A and Sudo A (2011) The symptom-to-diagnosis delay in soft tissue sarcoma influence the overall survival and the development of distant metastasis. J Surg Oncol, **104**(7):771-5 DOI: 10.1002/jso.22006.*
186. *Taylor WS, Grimer RJ, Carter SR, Tillman RM, Abudu A and Jeys L (2010) "Two-week waits"-are they leading to earlier diagnosis of soft-tissue sarcomas? Sarcoma, **2010** DOI: 10.1155/2010/312648.*
187. *Gerrand C, Francis M, Dennis N, et al. (2015) Routes to diagnosis for sarcoma - Describing the sarcoma patient journey. Eur J Surg Oncol, **41**(10):1393-9 DOI: 10.1016/j.ejso.2015.07.009.*
188. UK CR. Available from: https://www.cancerresearchuk.org/health-professional/cancer-statistics/statistics-by-cancer-type/soft-tissue-sarcoma.
189. *Fayet Y, Chevreau C, Decanter G, et al. (2022) No Geographical Inequalities in Survival for Sarcoma Patients in France: A Reference Networks' Outcome? Cancers (Basel), **14**(11) DOI: 10.3390/cancers14112620.*
190. *Martin-Broto J, Hindi N, Cruz J, et al. (2019) Relevance of Reference Centers in Sarcoma Care and Quality Item Evaluation: Results from the Prospective Registry of the Spanish Group for Research in Sarcoma (GEIS). Oncologist, **24**(6):e338-e346 DOI: 10.1634/theoncologist.2018-0121.*
191. *Trovik C, Bauer HCF, Styring E, et al. (2017) The Scandinavian Sarcoma Group Central Register: 6,000 patients after 25 years of monitoring of referral and treatment of extremity and trunk wall soft-tissue sarcoma. Acta Orthop, **88**(3):341-347 DOI: 10.1080/17453674.2017.1293441.*
192. *Jorgensen PH, Lausten GS and Pedersen AB (2016) The Danish Sarcoma Database. Clin Epidemiol, **8**:685-690 DOI: 10.2147/CLEP.S99495.*
193. *Van Meekeren M, Fiocco M, Ho VKY, Bovee J, Gelderblom H and Haas RL (2021) Patterns of Perioperative Treatment and Survival of Localized, Resected, Intermediate- or High-Grade Soft Tissue Sarcoma: A 2000-2017 Netherlands Cancer Registry Database Analysis. Sarcoma, **2021**:9976122 DOI: 10.1155/2021/9976122.*
194. *SWISS-SARCOMA*. Available from: https://www.swiss-sarcoma.net/.
195. *Cancer Trends - Soft tissue sarcomas*. Available from: https://www.ncri.ie/publications/cancer-trends-and-projections/cancer-trends-soft-tissue-sarcomas.

196. *Cancer of soft tissue without mesothelioma*. Available from: https://www.krebsdaten.de/Krebs/EN/Content/Cancer_sites/Soft_tissue_cancer_without_mesothelioma/soft_tissue_cancer_without_mesothelioma_node.html.
197. *Soft Tissue & Bone*. Available from: http://www.iccr-cancer.org/datasets/published-datasets/soft-tissue-bone.
